# Development, qualification, and validation of the Filovirus Animal Nonclinical Group anti-Ebola virus glycoprotein immunoglobulin G enzyme-linked immunosorbent assay for human serum samples

**DOI:** 10.1371/journal.pone.0215457

**Published:** 2019-04-18

**Authors:** Thomas L. Rudge, Karen A. Sankovich, Nancy A. Niemuth, Michael S. Anderson, Christopher S. Badorrek, Nick D. Skomrock, Chris M. Cirimotich, Carol L. Sabourin

**Affiliations:** 1 Battelle, West Jefferson, OH, United States of America; 2 Contract support for the U.S. Department of Defense (DOD) Joint Program Executive Office for Chemical, Biological, Radiological, and Nuclear Defense (JPEO-CBRND) Medical Countermeasures Systems Joint Vaccine Acquisition Program (MCS-JVAP), Fort Detrick, MD, United States of America; University of Texas Medical Branch at Galveston, UNITED STATES

## Abstract

The need for an efficacious vaccine against highly pathogenic filoviruses was reinforced by the recent and devastating 2014–2016 outbreak of Ebola virus (EBOV) disease in Guinea, Sierra Leone, and Liberia that resulted in more than 10,000 casualties. Such a vaccine would need to be vetted through a U.S. Food and Drug Administration (FDA) traditional, accelerated, or Animal Rule or similar European Medicines Agency (EMA) regulatory pathway. Under the FDA Animal Rule, vaccine-induced immune responses correlating with survival of non-human primates (NHPs), or another well-characterized animal model, following lethal EBOV challenge will need to be bridged to human immune response distributions in clinical trials. When possible, species-neutral methods are ideal for detection and bridging of these immune responses, such as methods to quantify anti-EBOV glycoprotein (GP) immunoglobulin G (IgG) antibodies. Further, any method that will be used to support advanced clinical and non-clinical trials will most likely require formal validation to assess suitability prior to use. Reported here is the development, qualification, and validation of a Filovirus Animal Nonclinical Group anti-EBOV GP IgG Enzyme-Linked Immunosorbent Assay (FANG anti-EBOV GP IgG ELISA) for testing human serum samples.

## Introduction

The filoviruses (family *Filoviridae*) from the genera *Ebolavirus* and *Marburgvirus* are etiologic agents of sporadic viral hemorrhagic fever outbreaks in humans with high mortality rates. An unprecedented outbreak of Ebola virus (EBOV; species *Zaire ebolavirus*) disease that began in Guinea during December 2013 [1] subsequently spread into neighboring West African countries of Sierra Leone and Liberia, prompting the World Health Organization (WHO) to declare the epidemic a public health emergency of international concern (http://www.who.int/mediacentre/news/statements/2014/ebola-20140808/en/). Phylogenetic analysis of viral isolates from this epidemic suggests a single transmission event introduced the virus, named the EBOV Makona variant [2], from an undetermined natural reservoir into humans in Guinea, followed by transmission between humans to spread the virus throughout Guinea and into Sierra Leone and Liberia [3]. Implementation of containment measures such as patient isolation and improved burial practices eventually controlled the epidemic, which resulted in 28,616 reported cases with a mortality rate of approximately 40% (http://www.who.int/csr/disease/ebola/en/).

The severity of this epidemic and principle transmission from human to human underscored the need for efficacious vaccines and therapeutics against EBOV, accelerating the placement of candidate EBOV vaccines into clinical safety trials [4–6]. Three vaccines have advanced into efficacy trials [4]: a chimpanzee adenovirus 3 (chAd3)-vectored vaccine [7, 8], a prime-boost combination vaccine vectored by human adenovirus 26 (hAd26) and Modified Vaccinia virus Ankara (MVA) [9], and a recombinant vesicular stomatitis virus (rVSV)-vectored vaccine [10, 11].

The EBOV glycoprotein (GP) is expressed on the exterior of the viral particle, is required for virus binding and entry into the cytoplasm of susceptible host cells, and is a primary target for neutralizing and protective antibodies [12–18]. During the 2014–2016 EBOV disease outbreak in Sierra Leone, higher levels of EBOV anti-GP-specific IgG at one week after the onset of symptoms were correlated with survival in 65 confirmed cases [19]. Therefore, an efficacious vaccine candidate likely would require strong induction of GP-specific antibodies. The vaccines that have been moved into clinical trials, and other published vaccines, present the EBOV GP as an antigen and induce anti-EBOV GP antibodies in non-human primates [20–25] and humans [4, 7–10, 26–37].

Unless clinical benefit can be directly demonstrated in human trials (e.g., during outbreaks), the efficacy of a filovirus vaccine or therapeutic will require evaluation under the FDA’s Animal Rule, as it is unethical to challenge humans in live EBOV studies. To implement this rule, immune responses that correlate with survival in well-characterized animal models must be bridged to the distribution of immune responses in placebo-controlled human clinical trials to establish human efficacy. Species-neutral immunological methods are ideal for bridging data between humans and animal models. Here, we describe the development of critical reagents and methods, qualification, and validation of the Filovirus Animal Nonclinical Group (FANG) [38] human anti-EBOV GP IgG ELISA for detection and quantitation of anti-EBOV GP IgG antibodies in human serum. The resulting ELISA method reproducibly quantifies levels of anti-EBOV GP IgG antibodies in human serum samples from EBOV disease survivors and vaccinated individuals.

## Results

### Anti-EBOV GP IgG ELISA development

To develop the anti-EBOV GP IgG ELISA, various parameters were optimized and critical reagents were generated. This was performed in a step-wise manner such that optimized parameters determined at each step were used in testing for all subsequent steps. First, the recombinant GP (rGP) coating concentration was optimized. Based on OD values obtained (S1 Fig), it was shown that the goat anti-human IgG conjugate binds human anti-GP IgG but does not bind directly to the rGP coating antigen, and the optimum rGP coating concentration was selected as 0.5 μg/mL (50 ng/well) and used for further assay development. Next, based on the OD values and binding ratios (S2 Fig), the optimal dilution of the conjugate was seleted to be 1:10,000 and used for further assay development.

The EBOV GP antibody-positive human serum Lot RMR1388D31 used for the antigen concentration and conjugate dilution optimizations showed high rGP-binding activity (based on OD values) and was selected as a candidate reference standard (RS) for the assay. Using the optimized rGP concentration and conjugate dilution, the generated 4-parameter logistic (4PL) curves at different starting dilutions showed similar curve shapes (S3 Fig); therefore, a starting dilution of 1:100 for the candidate RS was selected based on a maximum OD value of approximately 2.9 (S1 Table), which is in the ideal range of the microplate reader. Further characterization of the candidate RS using different serial dilution schemes (1:2, 1:1.8, 1:1.6, and 1:1.5) showed that a serial dilution of 1:1.5 when started at an initial dilution of 1:100 generated an optimal 4PL curve with the most dilution points in the linear range of the curve (S4 Fig). The candidate RS was arbitrarily assigned an antibody concentration of 1,000 ELISA units/mL.

Next, the quality control (QC) sera for the ELISA were generated and characterized. A candidate QC serum with high antibody concentration (QC-High; Lot number RMR1388D14) and QC serum with low antibody concentration (QC-Low; BMIZAIRE102) were evaluated, and the average concentrations were calculated to be 452.02 and 117.49 ELISA units/mL for the QC-High and QC-Low, respectively (S2 Table). Naïve human serum lots were then evaluated as candidate negative control (NC) sera for the ELISA. Ten unique lots of human serum were evaluated in the optimized ELISA. Seven of these lots generated OD values ≤0.10 (S5 Fig) and were pooled into a single volume that was designated as a candidate NC serum (Lot number BMI529).

### Anti-EBOV GP IgG ELISA qualification

#### Second- and third-generation control serum qualification

Due to limiting volumes of first-generation control sera containing anti-EBOV GP IgG (RS, QC-High, QC-Low), it was necessary to generate and qualify new lots of these critical reagents for use during the assay qualification and subsequent validation. The generation of these different lots is described in the Materials and Methods section. Each new lot of serum controls was qualified into the anti-EBOV GP IgG ELISA, with a summary of the results provided in Table 1.

**Table 1:** Summary of control serum lot qualifications.

Control Serum (Generation)	Lot Number	Concentration (ELISA Units/mL)	Acceptance Range (ELISA Units/mL)	Use^a^
RS (First)	RMR1388D31	1,000^b^	N/A	D/O
RS (Second)	BMIZAIRE102	1,009.46	N/A	Q, V
RS (Third)	BMIZAIRE108	876.22	N/A	V^c^
QC-High (First)	RMR1388D14	428.89	275.08–582.71	D/O
QC-High (Second)	BMIZAIRE103	582.53	356.42–808.64	Q, V
QC-High (Third)	BMIZAIRE110	490.37	293.01–687.73	V^c^
QC-Low (First)	BMIZAIRE102	115.90	59.38–172.42	D/O
QC-Low (Second)	BMIZAIRE104	148.40	78.34–218.46	Q, V
QC-Low (Third)	BMIZAIRE109	182.28	109.28–255.28	V^c^
NC (First)	BMI529	0	N/A	D/O, Q, V

a–D/O, Development/Optimization; Q, Qualification; V, Validation

b–value was arbitrarily assigned

c–third-generation sera were only used for evaluation of the ULOQ during assay validation

#### Qualification—General

Following optimization of the ELISA method and qualification of the critical reagents, ELISA performance was characterized through qualification and robustness testing. The ELISA limit of detection (LOD), limits of quantitation, repeatability, intermediate precision, and dilutional linearity were calculated from results obtained with 100 qualification test samples generated from 10 parent samples (range: 348.87–4662.37 ELISA units/mL) that were diluted in naïve human serum to various levels; the intent was to generate a panel of test samples spanning the dynamic range of the ELISA. The ELISA concentrations of each qualification test sample are provided in the supplemental information (S3 Table). An outlier analysis (S6 Fig) identified 14 potential outliers. Three of these data points were removed from the analyses due to ELISA concentrations being calculated from one or two dilution points. A summary of the qualification results are provided in Table 2.

**Table 2:** Summary of anti-EBOV GP IgG ELISA qualification results.

Parameter	Result
Negative Sample OD	95% Prediction Interval of 0.000–0.337
Limit of Detection	31.74 ELISA units/mL
Lower Limit of Quantitation	55.34 ELISA units/mL
Upper Limit of Quantitation	2,511.89 ELISA units/mL
Intermediate Precision	14.8% CV
Repeatability	8.2% CV
Dilutional Linearity (Accuracy)	Slope of -0.99 (90% CI of -1.03, -0.94)
rGP-Coated Plate Stability	7 days at 2–8°C
Serum Stability	21 days at 2–8°C 24 hours at room temperature 7 freeze/thaw cycles
rGP Stability	3 days at 2–8°C 8 freeze/thaw cycles (6 recommended)

#### Limit of background cutoff assessment

To generate an end-point titer cutoff value for antibody-negative samples in the ELISA, a mixed-effects analysis of variance (ANOVA) model was fitted to the average OD values from 150 naïve human serum samples tested in the ELISA (S4 Table). Prediction intervals at 95% and 99% confidence were determined to be 0.000–0.337 and 0.000–0.405 ELISA units/mL, respectively.

#### Qualification—Limits of detection

The LOD is the lowest antibody concentration for which there is at least 95% probability that an estimate can be obtained. From the logistic curve comparing the probability of detection against the predicted values for all test samples (S7 Fig), the LOD was estimated to be 31.74 ELISA units/mL, with a 95% confidence interval of 25.20–44.61 ELISA units/mL.

#### Qualification—Limits of quantitation

Quantitation limits for an assay establish the ranges in which dilutional linearity, intermediate precision, and repeatability are acceptable. The lower limit of quantitation (LLOQ) for the assay was determined to be 55.34 ELISA units/mL, and a conservative estimate for the upper limit of quantitation (ULOQ) was determined to be 2,511.89 ELISA units/mL. The ULOQ was set conservatively during qualification due to a lack of available human serum samples with high antibody concentrations for testing (>5,000 ELISA units/mL) and was re-evaluated during validation (see Validation–Limits of quantitation section) when such samples were available.

#### Qualification–relative accuracy via dilutional linearity

The accuracy of an analytical method describes how close the mean test results calculated by performing the procedure are to the true value of the analyte. The accuracy of the ELISA was characterized via dilutional linearity to evaluate whether the assay can obtain results that are proportional to the concentration of antibody in a given sample. Dilutional linearity was determined through comparison of the resulting value for a test sample to the spike level of each sample. The percent relative error was calculated for each test sample and dilution level (S5 Table), and only dilutions that met an arbitrary maximum desired percent relative error of 50% were used to evaluate dilutional linearity.

Under perfect dilutional linearity, the slope of the regression of log-transformed concentration on log-transformed spike level should be -1. The overall regression line across all test samples was -0.99 with a 90% confidence interval of -1.03 to -0.94, showing that the assay is accurate across the range of concentrations tested (S8 Fig).

#### Qualification—Precision

The precision of an analytical method describes the closeness of individual measures of an analyte when the procedure is applied repeatedly to multiple aliquots of a single homogeneous volume of a test sample. To evaluate the precision of the human anti-EBOV GP IgG ELISA, two parameters (intermediate precision and repeatability) were determined using results from the 100 qualification test samples (S3 Table). Intermediate precision incorporates variation due to operator-to-operator, day-to-day, and plate-to-plate; and the percent coefficient of variation (%CV) was calculated to be 14.8%. The %CV for repeatability was calculated to be 8.2%.

#### Robustness—rGP-coated plate stability

The stability of rGP-coated plates following storage at 2–8°C for up to seven days was evaluated using a panel of 10 human serum samples with known anti-EBOV GP IgG concentrations (S6 Table). The geometric mean ELISA concentrations and 95% confidence intervals for each serum sample under each condition are provided in the supplemental information (S7 Table). Based on the results, rGP-coated plates are considered stable for up to seven days storage at 2–8°C.

#### Robustness—Serum stability

The stability of serum samples stored at different conditions was evaluated using aliquots of the QC-High and QC-Low sera. Samples of each serum lot were evaluated in the ELISA following storage at 2–8°C for one, seven, 14, or 21 days or at room temperature for 24 hours, and after being subjected to one, three, five, or seven freeze/thaw cycles. The geometric mean ELISA concentrations and 95% confidence intervals for each serum under each condition are provided in the supplemental information (S8 Table). Both sera generated results consistent with baseline (Day 0) ELISA concentrations following storage at 2–8°C for up to 21 days or storage at room temperature for 24 hours and are considered to be stable for these storage conditions. Similarly, both sera generated results consistent with one freeze/thaw cycle when subjected to as many as seven cycles and are considered stable for these freeze/thaw conditions.

#### Robustness—rGP stability

The stability of rGP stored at 2–8°C for three, five, or seven days or following four, six, or eight freeze/thaw cycles prior to coating ELISA plates was evaluated using the proficiency panel of human serum samples also used for rGP-coated plate stability testing. The geometric mean ELISA concentrations and 95% confidence intervals for each of the proficiency panel samples are provided in the supplemental information (S9 Table). A random coefficients model fit to the 2–8°C storage results showed a positive slope that was statistically significant (p = 0.0003), indicating a trend for increasing ELISA concentrations with additional days of rGP storage at 2–8°C. Additionally, the variability of the concentrations obtained using rGP stored for five or seven days at 2–8°C was greater relative to Days 0 (baseline) or 3. Taken together, the results indicate that rGP is considered stable when stored at 2–8°C for up to three days.

The freeze/thaw results after four, six, and eight cycles were within the lower and upper acceptance criteria, although some variability was observed in results at eight freeze/thaws. Therefore, the rGP is considered stable for up to eight freeze/thaw cycles, but six or fewer is recommended.

### Parallelism

#### Parallelism between RS lots

Parallelism was evaluated between the three RS lots (RMR1388D31, BMIZAIRE102, and BMIZAIRE108) that have been qualified in the anti-EBOV GP-IgG ELISA. Parallelism between these sera, which were obtained from disparate sources, provides evidence to support the capability of the ELISA to reliably measure anti-EBOV GP IgG antibodies. For pair-wise comparisons, each RS lot was qualified by analyzing the new RS as a TS against the previous generation RS using a six-point dilution series; 96.9% of BMIZAIRE102 (tested against RMR1388D31) and 99.6% of BMIZAIRE108 RS (tested against BMIZAIRE102) TSs met the assay acceptance criteria for parallelism by the Plikaytis method [39]. For both RS lots, over 90% of the passing test samples displayed parallelism over four to six dilutions corresponding to an 8-fold to 32-fold range.

In addition, a linearized random coefficient model was fit to RS data from qualification, validation, and experimental assays in which the full eleven-point dilution series was used. Strong concordance between the three RSs was demonstrated, with lot-specific shifts from the overall slope of the model determined to be -0.61%, 1.87%, and -1.25% for RMR1388D31, BMIZAIRE102, and BMIZAIRE108, respectively (Table 3). These shifts are well within ±10%, indicating parallelism between the RSs with slight variation that can be attributed to plate-to-plate fluctuations.

**Table 3:** Estimated shift from the overall slope for each RS lot.

Reference Lot	Shift in Slope Relative to Overall Slope (%)		
	Mean	Lower Confidence Limit	Upper Confidence Limit
RMR1388D31	-0.61	-3.87	2.48
BMIZAIRE102	1.87	-0.18	5.20
BMIZAIRE108	-1.25	-4.37	1.49

#### Parallelism between RS lot BMIZAIRE102 and the WHO reference reagent 15/220

The parallelism between the second-generation RS (BMIZAIRE102) and the WHO Reference Reagent 15/220, serum from an American Red Cross convalescent patient that has been assigned an arbitrary concentration of 1 International unit (IU)/mL (sample 79; [40]), was evaluated. The 15/220 serum was diluted 1:10 in naïve human serum, to create Lot Number BMIZAIRE116. Using the anti-EBOV GP IgG ELISA, the concentration of BMIZAIRE116 was determined to be 2713.59 ELISA units/mL, thus, 1 IU/mL is equivalent to 27,135.90 ELISA units/mL. During parallelism assessment, 90.8% of the BMIZAIRE116 samples met the assay acceptance criteria by the Plikaytis method, and over 99% of the passing TSs displayed parallelism over four to six dilutions corresponding to an 8-fold to 32-fold range. Modified 4PL nonlinear regression models with sample-dependent EC_50_ were fitted to the RS and all passing BMIZAIRE116 TSs on each plate. Standardized residuals from these models were small (generally between -2 and 2), and there was no clear pattern in the residuals (S9 Fig). Thus, the residual analysis did not indicate any lack of fit to the model and suggests similarity between the two types of serum.

#### Parallelism between RSs and test samples

Parallelism between RS and TSs was evaluated for every TS using the assay acceptance criteria for parallelism based on the Plikaytis method. Twelve human samples spanning a broad range of concentrations were tested a total of 81 times over a two-month period; 95.1% met the assay acceptance criteria for parallelism and 75.3% displayed parallelism over four to six dilutions corresponding to an 8-fold to 32-fold range. Modified 4PL nonlinear regression models with sample-dependent EC_50_ were fitted to the RS and all passing test samples on each plate. Standardized residuals from these models were small (generally between -2 and 2), and there was no clear pattern in the residuals (S10 Fig). Thus, the residual analysis did not indicate any lack of fit to the model and suggests similarity between the human reference and test serum.

### Anti-EBOV GP IgG ELISA validation

Table 4 provides the validation parameters, corresponding acceptance criteria, and results for the detection of EBOV GP-specific IgG in human serum samples. The acceptance criteria were set based on results obtained during qualification of the assay. The antibody concentrations for each validation test sample (VTS) can be found in the supplemental information (S10 Table).

**Table 4:** Summary of anti-EBOV GP IgG ELISA acceptance and validation characteristics.

Parameter	Acceptance Criteria^a^	Observed Results from Assay Validation	Pass/Fail Status
Limit of Detection	Concentration: ≤50 ELISA Units/mL Endpoint titer: ≤100	Concentration: 27.14 ELISA Units/mL Endpoint titer: 100	Pass Pass
Lower Limit of Quantitation	Concentration: ≤75 ELISA Units/mL Endpoint titer: ≤200	Concentration: 66.96 ELISA Units/mL Endpoint titer: 100	Pass Pass
Upper Limit of Quantitation	N/A	Concentration: 55,526.77 ELISA Units/mL Endpoint titer: 36,800	N/A N/A
Intermediate Precision	Concentration: ≤25% CV Endpoint Titer: ≤50% CV	Concentration: 14.2% Endpoint titer: 27.9%	Pass Pass
Repeatability	Concentration: ≤20% CV Endpoint titer: ≥80% of samples with ≤10% difference ^b^	Concentration: 16.9% Endpoint titer: 100%	Pass Pass
Total Assay Variability	N/A	Concentration: 22.2% Endpoint titer: 39.2%	N/A N/A
Selectivity	Concentration: -35% to 35% Endpoint titer: 0.34 to 3.00	Concentration: -8.4% (range: -18.4% to 1.2%) Endpoint titer: 0.99 (range: 0.77 to 1.17)	Pass Pass
Interference			
Hemoglobin (High)	Concentration: -45% to 45% Endpoint titer: 0.34 to 3.00	Concentration: -2.4% (range: -9.8% to 7.1%) Endpoint titer: 1.05 (range: 0.93 to 1.20)	Pass Pass
Hemoglobin (Low)		Concentration: 0.9% (range: -0.5% to 4.2%) Endpoint titer: 1.06 (range: 1.00 to 1.20)	Pass Pass
Albumin		Concentration: 1.7% (range: -2.2% to 5.5%) Endpoint titer: 1.05 (range: 1.00 to 1.13)	Pass Pass
Triglycerides		Concentration: -4.0% (range: -15.9% to 12.0%) Endpoint titer: 1.16 (range: 1.00 to 1.63)	Pass Pass
Bilirubin		Concentration: 1.3% (-0.9% to 11.2%) Endpoint titer: 0.97 (range: 0.88 to 1.08)	Pass Pass
Specificity			
rGP (25 μg/mL)	Concentration: ≥3.7 Endpoint titer: ≥3.7	Concentration: 5.27 (90% lower bound: 4.47) Endpoint titer: 5.66 (90% lower bound: 4.65)	Pass Pass
CMV (25 μg/mL)	Concentration: ≤2.5 Endpoint titer: ≤2.5	Concentration: 1.03 (90% upper bound: 1.07) Endpoint titer: 0.99 (90% upper bound: 1.06)	Pass Pass
Dilutional Linearity			
- Slope (Spike Level)	Concentration: -1.20 to -0.80 Endpoint titer: -1.20 to -0.80	Concentration: -0.97 (range: -1.03 to -0.88) Endpoint titer: -0.96 (range: -1.07 to -0.84)	Pass Pass
- Slope (Starting dilution)	Concentration: -0.20 to 0.20 Endpoint titer: -0.20 to 0.20	Concentration: 0.00 (range: -0.04 to 0.05) Endpoint titer: -0.03 (range: -0.17 to 0.04)	Pass Pass

^a^–Acceptance criteria were determined during assay development and qualification.

^b^–Difference refers to a two-fold difference from the median of the test samples on a given plate

#### Validation—Limits of quantitation

The limits of quantitation for assay validation were calculated using the results obtained for test samples diluted at various spike levels to contain a broad range of antibody concentrations. For each sample, the spike levels at which percent total error was ≤50% or ≤60% for concentration or endpoint titer, respectively, at the upper and lower bounds of the tested range were identified. The final limits of quantitation were defined as the average of the concentrations or endpoint titers from the last spike level with acceptable accuracy. The LLOQ was determined to be 66.96 ELISA units/mL and an endpoint titer of 100. These values were within the established acceptance criteria of ≤75 ELISA units/mL and ≤200 for concentration and endpoint titer, respectively (Table 4). The percent total error at the first dilution steps (most-concentrated samples) were all less than the acceptable limits; therefore, the ULOQ for concentration was set at the maximum mean concentration across the test samples, which was 55,526.77 ELISA units/mL (Table 4). The ULOQ for endpoint titer was set at the maximum median titer determined across the three test specimens, 36,800 (Table 4). The ULOQs may be conservative estimates based on the range of samples tested during validation. No acceptance criteria were set for the ULOQ, as the assay is intended to detect the highest possible levels of antibody.

#### Validation—Limit of detection

**Fig 1:**
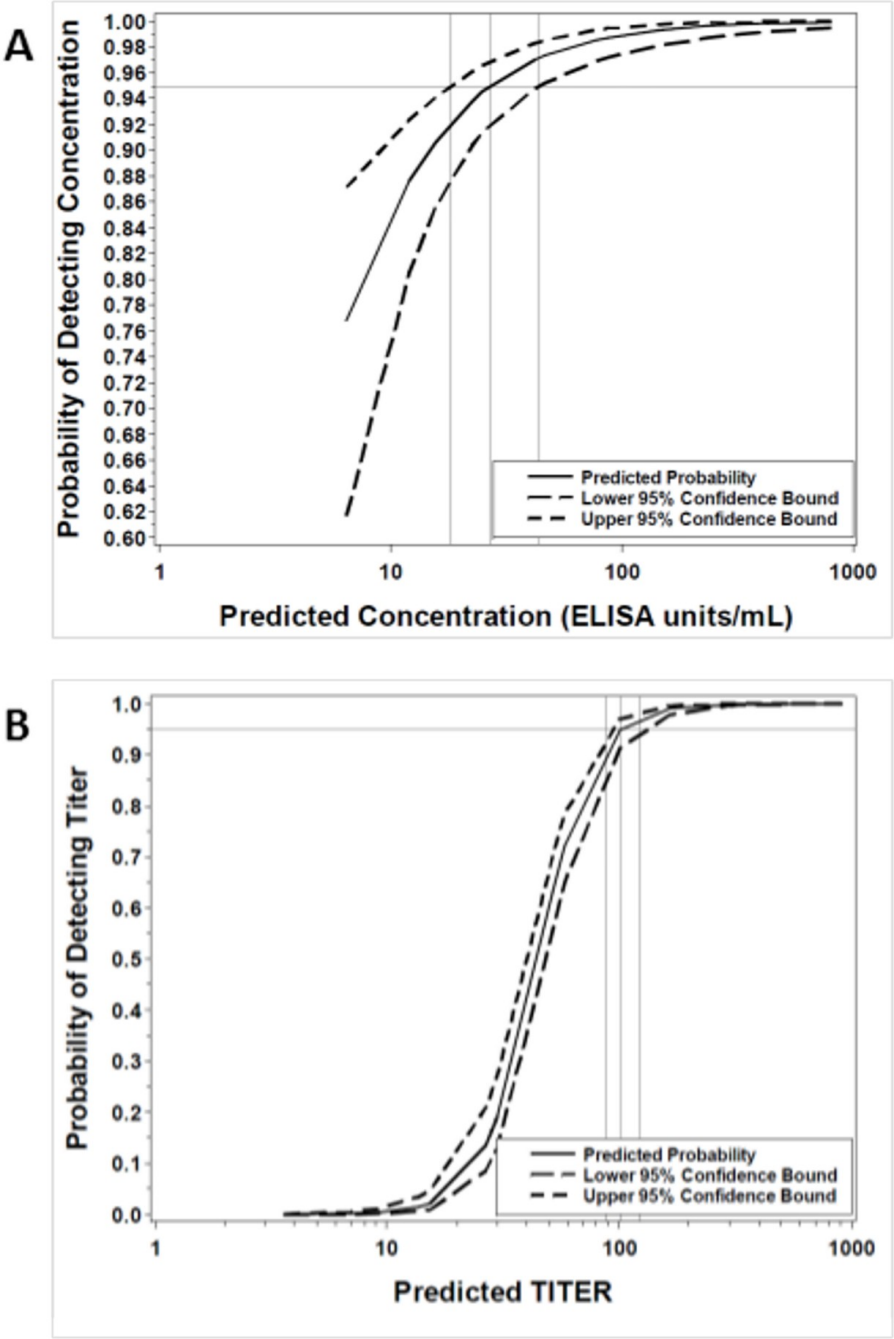
Estimated limits of detection. The logistic curves for the relationship between the probability of detecting the anti-EBOV GP IgG concentration (A) or endpoint titer (B) of a given sample and the predicted log-transformed value are represented by the solid lines, while dotted lines represent the upper and lower 95% confidence bounds for the estimate.

From the logistic curve comparing the probability of detection against the predicted values for all test samples, the LODs for concentration and endpoint titer were estimated to be 27.14 ELISA units/mL (Table 4, Fig 1A; 95% CI: 18.35 to 44.27 ELISA units/mL) and 101 (Table 4, Fig 1B; 95% CI: 88 to 123), respectively. Endpoint titer is a discrete value; therefore, a titer of 100 is the value closest to the estimate and is proposed as the LOD. These values passed the acceptance criteria of ≤50 ELISA unit/mL and ≤100 for concentration and endpoint titer, respectively (Table 4).

#### Validation–relative accuracy via dilutional linearity

For the human anti-EBOV GP IgG ELISA validation, accuracy was assessed by dilutional linearity to evaluate whether the assay could obtain results proportional to the antibody concentration or endpoint titer in test samples. The dilutional linearity of the assay was determined through comparison of the resulting value for a test sample to the spike level and to the starting dilution of each sample. Under perfect dilutional linearity, the slope of the regression of log-transformed concentration or endpoint titer on log-transformed spike level should be -1. Regression analysis of these values resulted in estimated slopes of -0.97 (90% CI: -0.99 to -0.95) and -0.96 (90% CI: -1.00 to -0.92) for concentration and endpoint titer, respectively; these values including the 90% CIs were entirely contained within the validation acceptance interval of -1.20 to -0.80 (Table 4). The dilutional linearity due to starting dilution, which should be zero under perfect dilutional linearity, also passed validation acceptance criteria. Regression analyses resulted in estimated slopes of 0.00 (90% CI: -0.03 to 0.02) and -0.03 (90% CI: -0.08 to 0.01) for concentration and endpoint titer, respectively, that were entirely contained within the acceptance interval of -0.20 to 0.20 (Table 4).

#### Validation—Precision

The operator-to-operator, day-to-day, and microplate-to-microplate variance components within the mixed model ANOVA contributed to the overall %CV for intermediate precision (Table 5), with the variation determined to be 14.2% and 27.9% for concentration and endpoint titer, respectively. The intermediate precision for concentration and endpoint passed the acceptance criteria of ≤25% and ≤50%, respectively (Table 4). The repeatability of the assay, based on a single analytical run performed by one operator using one set of equipment and consumables, was calculated to have a variation of 16.9% CV for concentration (Table 5), passing the acceptance criterion of ≤20% CV (Table 2). With respect to endpoint titer, 100% of repeatability VTSs had less than 10% of replicates on a given plate outside of a two-fold difference from their mean endpoint titer, passing the acceptance criteria of at least 80% of VTSs fitting these criteria (Table 4). The total assay variability, incorporating both intermediate precision and repeatability, was 22.2% and 39.2% for concentration and endpoint titer, respectively (Table 5). No acceptance criteria were set for total assay variability during this validation; however, this would be recommended for future validations.

**Table 5:** Repeatability and intermediate precision estimates.

Variance component	Concentration		Endpoint Titer	
	Variance	%CV	Variance	%CV
Operator	0.0009	6.9	0.0010	7.3
Day	0.0015	8.9	0.0059	17.8
Microplate	0.0014	8.6	0.0073	19.8
Residual (Repeatability)	0.0053	16.9	0.0128	26.5
Sum of Operator, Day, Microplate, Replicate Effects (Intermediate Precision)	0.0038	14.2	0.0142	27.9
Total Assay Variability Due to Method (Intermediate Precision and repeatability	0.0091	22.2	0.0269	39.2

#### Validation—Selectivity

The selectivity of the assay, or the impact of sample matrix variation on assay performance and the detection of anti-EBOV GP IgG antibodies, was evaluated by spiking individual or pooled naïve human sera with various concentrations of positive human immune serum. A highly-selective assay should be able to specifically detect anti-EBOV GP IgG antibodies; therefore, dilution of the antibodies in different matrix compositions should not impact the concentrations and endpoint titers calculated. In all conditions tested (three distinct positive human serum samples, each tested at two spike levels), the mean percent difference between the observed and expected concentration or endpoint titer was within the acceptance limits of -35% to 35% or 0.3 to 3.00, respectively. The mean percent difference in concentrations ranged from -18.4% to 1.2%, and the mean percent difference in endpoint titer ranged from 0.77 to 1.16 (Table 4).

#### Validation—Interference

To assess the ability of the assay to differentiate and quantify anti-EBOV GP IgG antibodies in the presence of other potential interferents normally encountered in human serum samples, four positive human serum samples were artificially spiked with hemoglobin (at two levels), albumin, triglycerides, or bilirubin. Comparison of the antibody levels in diluent-spiked samples to interferent-spiked samples provided a measure for the impact of each interferent on the specific detection of anti-EBOV GP IgG. When antibody concentration was evaluated in the test samples, the mean percent difference relative to mock-spiked samples for the five interferent scenarios ranged from -4.0% for the triglyceride-spiked samples to 1.7% for the albumin-spiked samples; all scenarios passed the acceptance criteria of -45% to 45% CV (Table 4). Similarly, all endpoint titers were calculated within the acceptance limits of 0.34 to 3.00, ranging from 0.97 for the bilirubin-spiked samples to 1.16 for the triglyceride-spiked samples (Table 4).

#### Validation—Specificity

The specificity of the assay was assessed by comparing calculated endpoints for ten positive human serum samples that were adsorbed with a homologous (EBOV rGP) or heterologous (CMV) antigen prior to analysis. The CMV antigen was selected because the CMV antigen is completely unrelated to filovirus antigens, CMV is highly prevalent in both African and North American populations and non-specificity would be of interest for further exploration [41, 42], and the recombinant protein was generated in a manner consistent with the EBOV rGP (expression in HEK293 cells). For a given serum sample, a decrease in the detectable anti-EBOV GP IgG levels when adsorbed with the rGP but no decrease when adsorbed with the CMV antigen indicated that the antibodies detected in the non-adsorbed sample were EBOV rGP-specific.

No change was observed in the test samples adsorbed with the CMV antigen, with observed mean ratios of anti-EBOV GP IgG in mock-adsorbed samples compared to samples adsorbed against the CMV antigen determined to be 1.03 and 0.99 (90% upper bound: 1.07 and 1.06) for concentration and endpoint titer, respectively. In contrast, the mean ratios for mock-adsorbed samples compared to adsorption with 25 μg/mL rGP were 5.27 and 5.66 (90% lower bound: 4.47 and 4.65) for concentration and endpoint titer, respectively. The CMV antigen- and 25 μg/mL rGP-adsorbed samples passed the validation acceptance criteria of ratios ≤2.5 and ≥3.7, respectively, in the corresponding 90% upper and lower bound (Table 4).

## Discussion

According to the FDA document entitled, “Guidance for Industry: Bioanalytical Method Validation,” validation of a bioanalytical method for quantitative measurement of analyte in a biological matrix, such as an ELISA, includes demonstrating the method is reliable and reproducible for the intended use [43]. In our validation, the FANG anti-EBOV GP IgG ELISA was found to be suitable for its intended purpose to measure human anti-EBOV GP IgG levels in human serum. In addition, the method is sensitive (low LOD), precise (intra- and inter-precision), dilutionally linear across its analytical range, selective (minimal matrix effects), and specific for anti-EBOV GP IgG in human serum as reported here. Two distinct assay endpoints were validated for the assay: concentration and endpoint titer. Both endpoints are valid as a final assay readout depending on the intended purpose; however, concentration is a more precise measurement due to its relative measure calculated based on the RS of the plate rather than the discrete and discontinuous nature of the endpoint titer. All parameters that were validated passed stringent criteria set for both endpoints through empirical testing and statistical analysis.

The progression of candidate EBOV vaccines through human clinical trials and to potential licensure via the FDA Animal Rule will be dependent on the bridging of immune correlates of protection from non-human primate (NHP) studies with equivalent human clinical trial endpoints. For example, previous NHP studies have suggested that anti-EBOV GP IgG antibody concentrations are a potential correlate of protection [19]. Successful bridging studies will be dependent on the development and validation of species-neutral methods (e.g., ELISAs, neutralization assays, etc.) for measuring these potential correlates in both humans and NHPs. To meet this demand, we have demonstrated here that the validated FANG anti-EBOV GP IgG ELISA is suitable for detecting human anti-EBOV GP IgG antibodies and can be used for assessing immunogenicity of candidate EBOV vaccines in human clinical trials or for assessing potential clinical cases.

## Materials and methods

### Recombinant Ebola virus (EBOV) glycoprotein (GP)

Purified rGP with amino acid sequence corresponding to the GP from EBOV Kikwit (Accession Number AFH89483.1) was produced at Advanced Bioscience Laboratories, Incorporated (Rockville, MD), Lot number 17OCT13, with a concentration of 1.53 mg/mL. The rGP is on a stability testing plan for total protein concentration and protein purity and has passed all stability criteria following storage at ≤-70°C for at least 36 months.

### Human reference standard serum

There have been three generations of reference standards (RSs) developed during the development/qualification and validation of the anti-GP IgG ELISA (Table 1). All samples were coded and no personally-identifiable information was provided. The authors did not interact with the subjects or have access to identifiable data. The use of these samples was evaluated by a representative of the Battelle Institutional Review Board and was determined not to meet regulatory criteria for categorization as human subjects research nor to require further IRB review, approval, and oversight. The first-generation RS (Lot Number RMR1388D31) was a single serum sample collected from an individual who received a needlestick from a potentially EBOV-infected needle. This individual received the experimental vaccine VSVΔG-ZEBOV two days post-needlestick [44], and serum used for the RS was collected 31 days post-vaccination and assigned an arbitrary concentration of 1000 ELISA units/mL. The second-generation RS (Lot Number BMIZAIRE102) was created by pooling serum from 25 human subjects that were vaccinated with VSVΔG-ZEBOV. This lot of RS was bridged to the RMR1388D31 lot and determined to have an anti-EBOV GP IgG concentration of 1,009 ELISA units/mL. The third-generation RS (Lot Number BMIZAIRE108) was created by pooling 592 distinct human serum samples from four different sources to generate a standard representative of three distinct vaccine platforms, VSVΔG-ZEBOV, Ad26.ZEBO + MVA-BN-Filo, and an EBOV GP nanoparticle vaccine. BMIZAIRE108 was deemed to be parallel with BMIZAIRE102 and was assigned an anti-EBOV GP IgG concentration of 876.22 ELISA units/mL.

The second-generation RS (BMIZAIRE102) was used for assay validation, except for evaluation of the upper limit of quantitation (ULOQ), which used the third-generation RS (BMIZAIRE108) after parallelism was established between the RS lots.

### Human quality control serum

There have been three generations each of quality control serum with high anti-EBOV GP IgG levels (QC-High) and quality control serum with low anti-EBOV GP IgG levels (QC-Low) generated during the development/qualification and validation of the assay (Table 1). All samples were coded and no personally-identifiable information was provided. The authors did not interact with the subjects or have access to identifiable data. The use of these samples was evaluated by a representative of the Battelle Institutional Review Board and was determined not to meet regulatory criteria for categorization as human subjects research nor to require further IRB review, approval, and oversight. The first-generation QC-High (Lot Number RMR1388D14) was a single serum sample from the same individual that received the VSVΔG-ZEBOV vaccine following a potential EBOV needlestick exposure (first-generation RS), collected 14 days post-vaccination and was determined to have an anti-EBOV GP IgG concentration of 428.89 ELISA units/mL [acceptance range (mean ± two standard deviations): 275.08 to 582.71 ELISA units/mL]. The second-generation QC-High (Lot Number BMIZAIRE103) was generated by first pooling positive serum from 14 humans vaccinated with VSVΔG-ZEBOV, followed by dilution in naïve human serum, and was qualified against the second-generation RS and determined to have an anti-EBOV GP IgG concentration of 547 ELISA units/mL [acceptance range (mean ± two standard deviations): 259.45 to 834.55 ELISA units/mL]. The third-generation QC-High (Lot Number BMIZAIRE110) was created by pooling serum from 21 humans vaccinated with VSVΔG-ZEBOV and was qualified against the second-generation RS and determined to have an anti-EBOV GP IgG concentration of 490.37 ELISA units/mL [acceptance range (mean ± two standard deviations): 293.01 to 687.73 ELISA units/mL].

The first-generation QC-Low (Lot Number BMIZAIRE101) was generated by diluting the first-generation QC-High serum 1:4 in naïve human serum and was determined to have an anti-EBOV GP IgG concentration of 115.90 ELISA units/mL [acceptance range (mean ± two standard deviations): 59.38 to 172.42 ELISA units/mL]. The second-generation QC-Low (Lot Number BMIZAIRE104) was generated by first pooling positive serum from 10 humans vaccinated with VSVΔG-ZEBOV, followed by dilution in naïve human serum and was qualified against the second-generation RS and determined to have an anti-EBOV GP IgG concentration of 148.4 ELISA units/mL [acceptance range (mean ± two standard deviations): 78.34 to 218.46 ELISA units/mL]. The third-generation QC-Low (Lot Number BMIZAIRE109) was created by pooling serum from 27 humans vaccinated with VSVΔG-ZEBOV and was qualified against the second-generation RS and determined to have an anti-EBOV GP IgG concentration of 182.28 ELISA units/mL [acceptance range (mean ± two standard deviations): 109.28 to 255.28 ELISA units/mL].

The second-generation QC sera were used for assay validation, except for evaluation of the ULOQ, which used the third-generation QC sera.

### Human negative control serum

The negative control (NC) serum (0 ELISA units/mL, Lot Number BMI530) was generated by pooling equivalent volumes of 23 individual naïve human serum samples purchased from Innovative Research (Novi, MI).

### Anti-EBOV GP IgG ELISA

The validated anti-*Bacillus anthracis* protective antigen IgG ELISA [45] was used as a template for the anti-EBOV GP IgG ELISA method, including the microtiter plates used, buffer compositions, incubation periods, peroxidase substrate, and stop solution. The ELISA procedure was performed by coating Immulon 2HB 96-well plates (Thermo Fisher Scientific, Waltham, MA) with rGP diluted in phosphate buffered saline (PBS) (optimized concentration of 0.5 μg/mL (50 ng/well)). Plates were incubated overnight at 2–8°C and then washed three times with Wash Buffer (PBS plus 0.1% Tween-20). For each plate, control samples or test samples (TSs) diluted in ELISA Diluent (PBS plus 5% skim milk and 0.1% Tween-20) were added in 100 μL or 200 μL volumes, respectively, as follows: Columns 1-11/Rows A and B were loaded with the RS dilutions and Column 12/Rows A and B were loaded with the negative control serum. The TSs (10) were loaded into Columns 1-10/Row C, and the QC-High and QC-Low serum samples were loaded into Columns 11-12/Row C. A six-point, two-fold dilution series of each TS and QC sample was then generated in ELISA Diluent by serial transfer of 100 μL down the plate, with 100 μL discarded from the bottom row. The plate was incubated at 37°C for one hour. ELISA Diluent contains skim milk; therefore, an additional blocking step is not required for the assay.

Following incubation, the plate was washed three times with Wash Buffer. Horseradish peroxidase (HRP)-conjugated goat anti-human IgG (Fcγ fragment-specific; Jackson ImmunoResearch Laboratories, Inc.; West Grove, PA) diluted in ELISA Diluent (optimized dilution of 1:10,000) was added to all wells (100 μL/well), and the plate was incubated at 37°C for 1 hour. The plate was then washed five times with Wash Buffer, and 100 μL of room temperature-equilibrated TMB Substrate (Thermo Fisher Scientific) was added to all wells. Following incubation at room temperature for an additional 10 min, 100 μL of TMB Stop Solution (Thermo Fisher Scientific) was added to all wells. Within 30 min of TMB Stop Solution addition, optical density (OD) readings for each well were obtained at 450 nm with a reference wavelength of 650 nm using a BioTek ELx800 microplate reader (BioTek, Winooski, VT) accompanied by Gen5 software.

To calculate a reportable anti-EBOV GP IgG concentration for a sample, a 4-Parameter Logistic (4PL) curve was first fit to the OD values obtained for the RS. The four parameters of the 4PL model are the lower asymptote of the sigmoidal curve (*a*), a curvature parameter related to the slope of the curve (*b*), a parameter related to the dilution at the midpoint of the curve (*c*), and the upper asymptote of the curve (*d*). The concentrations (based on the acceptance criteria delineated below) from each dilution of the six-point dilution series of a TS were then calculated from the RS using the 4PL equation. The reported concentration for each TS was the arithmetic average of the values from the acceptable dilution points.

For the validation, the endpoint titer was calculated as the inverse of the dilution of the first dilution point of the TS dilution series above the upper limit of the 95% prediction interval, which is 0.337 for the human assay.

### ELISA development and optimization

The rGP coating concentration was optimized by evaluating rGP concentrations of 0.3 to 1.0 μg/mL diluted in 1X phosphate buffered saline (PBS) with the first-generation RS serum RMR1388D31 and a naïve human serum sample (Sigma). The various rGP concentrations were coated onto a microtiter plate, one concentration per row, and a two-fold dilution series of the RS at a starting dilution of 1:50 and a single point of the naïve serum at 1:50 dilution were evaluated. The OD values from the RS wells were used to generate Four Parameter Logistic (4PL) curves for each rGP concentration.

The HRP-conjugated goat anti-human IgG antibody conjugate dilution was optimized using a checkerboard titration strategy in which conjugate dilutions from 1:6,000 to 1:12,000 were evaluated in separate rows of a microtiter plate, and dilutions of the first-generation RS and naïve sera were evaluated in the columns of the plate. The optimal rGP coating concentration of 0.5 μg/mL (50 ng/well) was used. OD values were used to calculate binding ratios by dividing the OD values from the RS serum by the corresponding OD values for the naïve serum.

The first-generation RS starting dilution and serial dilution scheme were evaluated using the optimized rGP coating concentration and conjugate dilution. Starting dilutions of RS serum RMR1388D31 of 1:50, 1:80, 1:100, and 1:200 and serial dilution schemes of 1:2, 1:1.8, 1:1.6, and 1:1.5 were evaluated, and the resulting OD values were used to generate RS curves from a 4PL model.

The first-generation QC-High (Lot number RMR1388D14) was diluted 1:4 in naïve human serum to generate a candidate QC-Low (Lot number BMIZIARE101). These QC sera were evaluated at an initial 1:50 dilution with a 1:2 serial dilution scheme using the optimized parameters and the RS assayed under optimized conditions. The concentrations for each QC-High and QC-Low replicate were determined using the RS curve.

Additional lots of naïve human serum were evaluated as candidate NC for the ELISA. A total of 10 human serum lots (Innovative Research, Inc.) were evaluated at a starting dilution of 1:50 in the optimized assay.

### ELISA qualification

#### Serum control qualification

Prior to qualification of the ELISA, the QC serum controls used in the qualification (Table 1) were characterized by evaluating them multiple times in the ELISA to generate acceptance ranges around the ELISA concentrations. Results from evaluating the QC-High serum Lot Number BMIZAIRE103 either as a test sample or as a QC a total of 43 times were used to generate the preliminary acceptance range, which was updated and finalized following 820 independent results for this serum. Results from evaluating the QC-Low serum Lot number BMIZAIRE104 either as a test sample or as a QC a total of 56 times were used to generate the preliminary acceptance range, which was updated and finalized following 775 independent results for this serum.

#### Naïve serum ELISA prediction intervals

A total of 150 naïve human serum samples (Innovative Research, Inc and Focus Diagnostics, Inc., Cypress, CA) were evaluated in duplicate at a single 1:50 dilution in the optimized ELISA. The arithmetic average OD value for each sample was calculated, and a mixed-effects ANOVA model was fitted to the untransformed average values. The model was used to develop 95% and 99% prediction intervals.

#### Qualification test samples

An initial panel of 10 human serum samples positive for anti-EBOV GP IgG (Table 6; approximate concentration range: 348.84 to 4662.37 ELISA units/mL) were diluted in NC serum Lot number BMI529 to various levels to generate a total of 100 qualification test samples (QTSs) used for the qualification testing (S11 Table). All parent samples were coded and no personally-identifiable information was provided. The authors did not interact with the subjects or have access to identifiable data. The use of these samples was evaluated by a representative of the Battelle Institutional Review Board and was determined not to meet regulatory criteria for categorization as human subjects research nor to require further IRB review, approval, and oversight.

**Table 6:** Immune human serum samples used to generate QTSs.

Serum ID	Approximate Concentration (ELISA Units/mL)
BMIZAIRE105	1157.22
3343.2163-005.D14	1425.20
3343.2163-006.D14	348.84
3343.2163-010.D14	373.41
3343.2163-013.D180	1031.23
3343.2163-016.D180	1905.90
3343.2163-023.D84	2360.47
3343.2163-024.D84	4136.22
3343.2163-034.D84	4662.37
3343.2163-071.D28	3279.49

The original positive serum samples were obtained from a candidate EBOV GP-based vaccine Phase I clinical trial conducted in the U.S., and were determined to be positive for anti-EBOV GP IgG using the ELISA assay described here. Prior to the performance of qualification testing, the samples were prepared, separated into two approximately equal-volume aliquots, and stored at ≤-70°C until use. Each QTS was evaluated at least twice by each of four individual operators in pre-determined plate layouts.

#### Qualification statistical analyses

ELISA concentrations were calculated for each test sample run. Results were log-transformed, and a preliminary outlier analysis was performed. Once three identified outliers were removed, the following statistical analyses were performed:

***Dilutional linearity*:** A random regression model was fit to results from all 10 dilutions for each parent test sample to relate the log-transformed ELISA concentration to the log-transformed final dilution of each test sample. The percent total error for each test sample and dilution level was then calculated from the model and back-transformed to the observational scale. An arbitrary cutoff of 50% was used as the desired maximum percent relative error; therefore, a similar random regression model was refit to results from each test sample using only those dilution levels where the percent relative error was less than 50%. The slope and corresponding 90% condifence interval for each test sample and the overall regression line across all 10 parent test samples were calculated.

***Limit of detection*:** A logistic regression analysis was performed to predict the probability that the ELISA concentration could be determined as a function of its predicted log-transformed concentration. The LOD was then estimated from the model as the lowest predicted concentration with at least 95% probability of determination.

***Limits of quantitation*:** The ULOQ and LLOQ represent the concentrations that bound the range of values for which dilutional linearity and precision are demonstrated. The ULOQ was set using the random regression model generated for dilutional linearity evaluation. The final LLOQ was calculated as the average of the 10 corresponding averages from the last dilution level with acceptable dilutional linearity.

***Precision*:** To assess intermediate precision and repeatability, observations with predicted ELISA concentrations greater than or equal to the calculated LLOQ were used to generate a mixed effect ANOVA model that included fixed effects for log-transformed dilution and parent test sample, and random effects for operator, day, plate-to-plate, and replicate within plate. The estimated variance components from the model were then used to calculate repeatability and intermediate precision, expressed as the percent coefficient of variation (CV) across all test samples above the LLOQ. The %CV for each source of variance was calculated as $100\times \sqrt{e^{\ln{\left(10\right)}^{2}\times \sigma^{2}}-1}$ where σ^2^ is the model-estimated variance for the specific variance source. The %CV for the intermediate precision of the assay was calculated as $100\times \sqrt{e^{\ln{\left(10\right)}^{2}\times \sigma_{T}^{2}}-1}$ where $\sigma_{T}^{2}$ is the sum of the model-estimated variances for day, operator, and plate. The %CV associated with the replicate effect served as an estimate for the assay repeatability.

### ELISA robustness

#### rGP-coated plate stability

In order to evaluate the stability of rGP-coated plates, a proficiency panel of 10 serum samples was developed. A parent serum sample was generated by first pooling volumes of the same serum used as parent samples for the qualification testing. This sample was determined to have a concentration of 4199.87 ELISA units/mL and was diluted 1:4 in NC serum Lot number BMI529. The resulting sample, Lot number BMIZAIRE105a, was determined to have a concentration of 1031 ELISA units/mL. The 10-sample panel was then created by dilution of BMIZAIRE105a in BMI529 to target levels indicated in S6 Table.

Test plates for the stability testing were coated with rGP at 0.5 μg/mL and were stored at 2–8°C for one, three, five, or seven days (four plates per timepoint). At each timepoint, four individual operators assayed one plate in order to directly compare each timepoint to the baseline condition of 1 day at 2–8°C. Each operator assayed the 10-serum proficiency panel on each plate. For statistical analysis of the resulting ELISA concentrations, a random coefficients model was fit to the plate stability data. The response variable for the model was the base-10 log-transformed concentration, which was regressed against the number of coating days. The human proficiency panel samples (test samples) were treated as a random effect in the model. In a random coefficients model, the intercept and slope of the fitted regression line are assumed to be a random sample from some population of possible coefficients. Model residuals were examined to assess the assumption of data normality and to identify potential outliers. The regression coefficients were estimated, and the slope of the overall fitted regression line was tested to determine if it was significantly different from zero. The geometric mean concentration at the baseline (Day 1) as well as the geometric mean concentration and corresponding 95% confidence bounds at each of Days three, five, and seven were predicted from the model for each proficiency panel sample. An arbitrary acceptance criterion of 30% was used to assess stability, where the 95% confidence bounds of the fitted regression line must be within the acceptance criteria in order to consider the plates at the timepoint to be stable for use in the ELISA.

#### Human serum stability

Human serum stability at different storage conditions was evaluated using the QC-High serum Lot BMIZAIRE103 and QC-Low serum Lot BMIZAIRE104. Aliquots of each serum were subjected to the following storage conditions: storage at 2–8°C for zero, one, seven, 14, or 21 days or storage at room temperature for 24 ± 8 hours. Aliquots of these sera were also subjected to one, three, five, or seven freeze/thaw cycles, with a cycle consisting of freezing the serum at ≤ -70°C followed by a thaw at room temperature for 30 minutes. Serum aliquots were evaluated in the optimized ELISA in triplicate for each storage condition by each of two test operators. A random coefficients model was fit to the resulting data for storage conditions or freeze/thaw stability in a manner similar to the model described for the rGP-coated plate stability testing. The geometric mean concentration at the baseline (Day 0 for storage or one freeze/thaw cycle) as well as the geometric mean concentration and corresponding 95% confidence bounds at each storage day or freeze/thaw were predicted from the model for each QC sample. An arbitrary acceptance criterion of 30% was used to assess serum stability, where the 95% confidence bounds of the fitted regression line must be within the acceptance criteria in order to consider the serum under the storage condition or freeze/thaw cycle to be stable for use in the ELISA.

#### rGP stability

The stability of rGP was examined under the following conditions: two, four, six, or eight freeze/thaw cycles (with a cycle consisting of freezing the serum at ≤ -70°C followed by a thaw at room temperature for 30 minutes) or storage at 2–8°C for three, five, or seven days. The rGP was subjected to these conditions and then used to coat plates for the ELISA at 0.5 μg/mL. The proficiency panel used for rGP-coated plate stability testing was also used for this testing (S6 Table). A random coefficients model was fit to the resulting data for rGP storage conditions or freeze/thaw stability in a manner similar to the model described for the rGP-coated plate stability testing. The geometric mean concentration at the baseline (Day 0 for storage or one freeze/thaw cycle) as well as the geometric mean concentration and corresponding 95% confidence bounds at each storage day or freeze/thaw were predicted from the model for each QC sample. An arbitrary acceptance criterion of 30% was used to assess rGP stability, where the 95% confidence bounds of the fitted regression line must be within the acceptance criteria in order to consider the rGP under the storage condition or freeze/thaw cycle to be stable for use in the ELISA.

### ELISA acceptance criteria

The anti-EBOV GP IgG ELISA Standard Operating Procedure used for validation has acceptance criteria that were defined during assay development and qualification. These criteria must be met to use the generated data for statistical analysis. These criteria are based on the acceptance of an entire microplate assay and the acceptance of an individual TS, as described below.

### Plate acceptance criteria

The plate acceptance criteria are based on the evaluation of the NC, RS, QC-High, and QC-Low values and are used to determine whether the entire microplate meets the minimum standards evaluated during assay development. If any one of these criteria fail, the entire plate fails. These criteria include the following:

***NC***: The OD value for the NC must be ≤0.20.

***RS***: For the RS acceptance criteria, the percent relative error (%RE) of individual dilution pairs within the linear range of the RS standard curve are evaluated. The linear range is determined by first identifying anchor points; the lower anchor point is defined as the low concentration point where the mean OD value of the dilution pair is <0.20 (the maximum allowable mean OD value for the NC) and the high anchor point is defined as the high concentration point where the OD fit to the 4PL model for the known concentration of the dilution point is >0.90 times the upper asymptote from the model (0.9 x *d*). All remaining RS dilution pairs are considered in the linear range of the standard curve and are used for evaluating the RS acceptance criteria. There must be at least five data points included in the linear range of the curve and at least 75% of these dilution points must have a %RE ≤20%.

***QCs***: Each of the QCs (QC-High and QC-Low) must have calculated anti-GP IgG concentrations within the predetermined acceptance ranges for each of the QCs used. For the performance of assay validation in the current study, the acceptance ranges for the QC-High and QC-Low were 259.45–834.55 and 78.34–218.46 ELISA units/mL, respectively.

### Test sample acceptance criteria

The TS acceptance criteria are based on the evaluation of the dilution series from individual test samples to ensure the minimal criteria are met. Failure of one or more TSs on a single microplate does not impact the pass/fail status of other samples on the same plate.

For a TS dilution to be accepted for evaluation of overall sample acceptance, the following criteria must be met: 1) the observed OD value must fall within the RS range (highest and lowest OD values of the RS dilution series); 2) the observed OD value must be greater than the mean OD value for the NC; and 3) the within-assay %CV must be ≤ 20%. Test sample dilutions that do not pass these criteria are excluded from analysis. The dilutions that pass the criteria are then used to calculate the anti-EBOV GP IgG concentration for the TS. Test samples that pass all acceptance criteria and have an OD value of the highest-concentrated (1:50) dilution less than the NC cutoff were considered to have passed and assigned a result of 0.00 ELISA units/mL. Test samples that did not pass the TS acceptance criteria were repeated.

### Evaluation of parallelism

During assay development and qualification, statistical analyses were performed to evaluate parallelism to ensure the calculated anti-EBOV GP IgG concentrations are consistent across the range of dilutions tested for TSs and RSs.

Three statistical approaches were used to evaluate parallelism: the Plikaytis method [39], which relies on consistency of the calculated concentration at multiple dilutions; a variation on the 4PL regression model; and random coefficients models fitted to transformed OD and concentration values.

The Plikaytis method requires that the percent coefficient of variation (%CV) of the anti-EBOV GP IgG concentration over at least three dilutions be less than 20%. Test samples meeting the Plikaytis method %CV criteria are considered sufficiently parallel to the RS to provide a reliable estimate of the anti-GP IgG concentration. A high proportion of TSs meeting the %CV criteria is indicative that the TS and RS are parallel. The number of dilutions over which samples met the %CV criteria was also considered, as more dilutions indicate parallelism over a broader range. For the 2-fold dilution series used in the EBOV anti-GP IgG ELISA, the minimum of three dilutions indicates parallelism over a 4-fold range, while the maximum of six dilutions indicates parallelism over a 32-fold range.

Parallelism between the RS and the TS and QC samples on the same plate was evaluated by fitting a modified 4PL model with sample-dependent half maximal effective concentration (EC_50_; the *c* parameter of the 4PL model) to the mean OD values against the base-10 log of the relative concentration. To determine parallelism, the modified 4PL model was fit individually for each plate to the RS and all passing TS and QC samples, under the assumption that the RS and all samples had the same upper and lower asymptotes and slope parameters, but allowing the TS EC_50_ concentration to vary. The model was evaluated by calculating standardized residuals for the model on each plate and visually assessing the standardized residuals across all plates in a given data set.

To evaluate parallelism over the three generations of RS, random coefficients linear regression models were used to model the relationship between log-transformed OD and log concentration for randomly selected RS curves. The models included an overall slope and intercept and RS-specific shifts from the overall slope and intercept. A bootstrap procedure was used to estimate 90% confidence intervals for the shift in slope relative to the overall slope.

### Validation test samples

An initial panel of 55 human serum samples positive for anti-EBOV GP IgG (Table 7; approximate concentration range: 79 to 45,418 ELISA units/mL), and six anti-EBOV GP IgG-negative samples (Table 8) were used to generate a total of 232 validation test samples (VTSs) used for a majority of the validation testing (S12 Table). Twenty-five (25) real-world (incurred) samples were also used to evaluate repeatability and precision. All samples were coded and no personally-identifiable information was provided. The authors did not interact with the subjects or have access to identifiable data. The use of these samples was evaluated by a representative of the Battelle Institutional Review Board and was determined not to meet regulatory criteria for categorization as human subjects research nor to require further IRB review, approval, and oversight.

**Table 7:** Immune human serum samples used to generate VTSs.

Serum ID	Category of VTS	Approximate Concentration (ELISA Units/mL)	Pool or Individual
W092115060052-A	Specificity	3218.36	Individual
W092115060052-B	Real World	3354.18	Individual
W092115060057-A	Dilution Linearity	3056.66	Individual
W092115060057-B	Specificity	3387.57	Individual
W092115060064-A	Dilution Linearity	2608.48	Individual
W092115060064-B	Real World	2665.73	Individual
W092115060071-A	Matrix Effects	4184.73	Individual
W092115060101-B	Real World	2144.02	Individual
W092115060101-C	Interference	2274.24	Individual
W092115060138-A	Real World	2184.45	Individual
650050315424	Real World	4806.33	Individual
650062386624	Dilution Linearity	4203.13	Individual
650041667024	Specificity	2891.78	Individual
650062385224	Specificity	7019.07	Individual
650062384124	Matrix Effects	7038.11	Individual
650041667124	Interference	2974.86	Individual
650062384524	Real World	3542.14	Individual
650062383424	Dilution Linearity	11948.49	Individual
650062383724	Real World	6400.95	Individual
650062384224	Real World	6912.47	Individual
10113168–03	Real World	179.15	Individual
10113528–03	Real World	129.17	Individual
10114015–03	Dilutional Linearity	3166.36	Individual
10113989–03	Real World	716.98	Individual
10130517–03	Real World	493.88	Individual
10113200–03	Dilution Linearity	1751.83	Individual
10113735–03	Specificity	2017.33	Individual
BMIZAIRE114	Interference	2066.08	Pool
10114436–03	Specificity	1335.25	Individual
10131804–03	Matrix Effects	17981.53	Individual
3344.008.D14	Real World	86.41	Individual
3344.011.D180	Real World	84.58	Individual
3344.013.D180	Dilution Linearity	2995.63	Individual
3344.014.D14	Real World	319.46	Individual
3344.020.D56	Real World	190.02	Individual
3344.025.D14	Real World	100.34	Individual
3344.037.D14	Real World	79.09	Individual
3344.042.D56	Specificity	2499.05	Individual
3344.043.D84	Dilution Linearity	4277.53	Individual
3344.044.D84	Specificity	1252.50	Individual
C1703024660	Real World	702.37	Individual
C1703090460	Specificity	800.02	Individual
C1703089620	Real World	171.35	Individual
BMIZAIRE112	Dilutional Linearity	9294.91	Pool
C1703087970	Specificity	900.00	Individual
C1703186220	Real World	300.40	Individual
C1703042080	Real World	79.18	Individual
BMIZAIRE113	Dilutional Linearity	5391.89	Pool
C1703060550	Real World	131.02	Individual
C1703068810	Real World	225.66	Individual
C1703187960	Real World	405.51	Individual
BMIZAIRE117	ULOQ	45,417.72	Pool
BMIZAIRE118	ULOQ	40,875.24	Pool
BMIZAIRE119	ULOQ	40,323.33	Pool

**Table 8:** Naïve human serum samples used to generate VTSs.

Sample ID	Pooled or Individual
BMI530	Pooled
23 79329	Individual, male
23 79327	Individual, male
88 21595	Individual, female
88 22472	Individual, female
88 22458	Individual, female

The original positive serum samples were obtained from multiple candidate EBOV GP-based vaccine Phase I clinical trials conducted in the U.S., Australia, and Canada and were determined to be positive for anti-EBOV GP IgG using the ELISA assay described here. Pooled or individual sera obtained from Innovative Research were used as the negative sera and were determined using this assay to have concentrations of 0 ELISA units/mL. Prior to the performance of validation testing, the samples were prepared, separated into four approximately equal-volume aliquots, and stored at ≤70°C until use. In general, VTSs were evaluated eight times, twice by each of four individual operators. Exceptions were samples tested as part of the repeatability and intermediate precision evaluations, which were tested 16 times total by the four operators, and samples tested as part of the ULOQ evaluation, which were tested either 12 or 20 times total by four operators.

***Dilutional Linearity Samples***: The dilutional linearity samples (VTS 1–100) were generated by differentially diluting ten samples positive for anti-GP IgG antibodies into naïve serum to generate a 7-step dilution series (eight VTSs per positive sample) for each positive sample (80 VTSs total). Ten of these VTSs were used for starting dilution replication; that is, one VTS was tested at the indicated starting dilution plus two additional starting dilutions (2X and 0.5X). These VTSs were used for the evaluation of limit of detection (LOD), relative accuracy via dilutional linearity, precision, and limits of quantification. The positive samples had expected anti-GP IgG concentrations ranging from 1,752 to 11,948 ELISA units/mL.

***Matrix Effects/Selectivity Samples***: The matrix effect VTSs (VTS 101–136) were generated from three positive human serum samples spiked at two dilutions (1:5 and 1:50) into five individual naïve human serum samples and into one pooled naïve human serum sample (36 VTSs total).

***Interference Samples***: The interference VTSs (VTS 137–172) were generated by spiking a known interferent into human serum samples. For this assessment, one of five interferents (high hemoglobin, low hemoglobin, albumin, triglycerides, or bilirubin) was spiked into one of three positive human serum samples or one naïve human serum sample. The sera containing interferents and matching mock samples containing no interferent were prepared as follows:

*High hemoglobin concentration (7*.*13 mg/mL)*: Hemoglobin levels in hemolytic serum (BioreclamationIVT; Westbury, NY) were measured using a validated Advia 120 Hematology Analyzer (Siemens, Deerfield, IL). Neat human serum samples were then diluted at 1:20 in hemolytic serum to generate a final hemoglobin concentration of 7.13 mg/mL. Mock samples were generated by diluting neat human serum samples at 1:20 in negative control human serum. *Low hemoglobin concentration (1*.*5 mg/mL)*: Neat human serum and hemolytic serum at final dilutions of 1:20 and 1:5, respectively, were diluted in negative control human serum, for a final hemoglobin concentration of 1.5 mg/mL. The anti-GP IgG concentration in the resulting solutions was equivalent to the anti-GP IgG concentrations in the high hemoglobin concentration samples, so the same mock sample was used for both. *Albumin (50 mg/mL)*: Albumin powder (Sigma-Aldrich Corp.; St. Louis, MO) was mixed with neat human serum to a final concentration of 50 mg/mL. Mock samples were prepared by combining neat human serum and negative control serum in volumes equivalent to the neat human serum and albumin in the test samples. *Triglycerides (5 mg/mL)*: A 20X triglyceride mix (100 mg/mL; Sigma-Aldrich) was diluted at 1:20 in neat human serum. Mock samples were prepared by diluting negative control serum at 1:20 in the neat human serum. *Bilirubin (0*.*15 mg/mL)*: On the day of testing, a 3 mg/mL solution of bilirubin (Sigma-Aldrich) was prepared in DMSO, then diluted at 1:20 in neat human serum. Mock samples were prepared by diluting DMSO at 1:20 in neat human serum.

***Specificity Samples***: The specificity VTSs (VTS 173–212) were generated by adsorbing 10 individual positive human serum samples with rGP antigen (25 μg/mL), human cytomegalovirus (CMV) glycoprotein antigen (25 μg/mL; GlaxoSmithKline), or ELISA Diluent (Mock). Antigens were first diluted in ELISA Diluent to the appropriate concentration, then neat human serum samples were diluted at 1:50 in the antigen solution. Mock samples were prepared by diluting neat human serum samples at 1:50 in ELISA Diluent. All samples were then incubated at 37°C for 60 minutes before being evaluated in the assay.

***Real World (Incurred) Samples*:** The real world (incurred) VTSs (VTS 203–227) were obtained from vaccinated individuals in Phase I clinical trials and were not diluted prior to analysis. The samples represent positive human serum samples typically seen during clinical testing and span the range of the ELISA. There were five, six, four, five, and five samples with anti-EBOV GP IgG concentrations in the 50–100, 100–250, 250–500, 500–3,000, and greater than 3,000 ELISA units/mL ranges, respectively.

***ULOQ Samples*:** The ULOQ VTSs (VTS 228–257) were generated by differentially diluting three samples with high anti-EBOV GP IgG antibody titers into naïve serum to generate a 9-step dilution series (10 VTSs per positive sample) for each positive sample (30 VTSs total). These VTSs were used for the evaluation of the ULOQ. The positive samples had expected anti-GP IgG concentrations ranging from 1,321 to 45,418 ELISA units/mL based on limited pre-validation testing.

### Assay validation

#### Test sample endpoints

For each VTS evaluated during assay validation, two assay endpoints were reported: concentration in ELISA units/mL and endpoint titer. Analysis of both endpoints was performed in a similar manner for each validation parameter, such as log transformation of the data. Validation acceptance criteria were based on both endpoints (Table 4).

#### Dilutional linearity

For the anti-EBOV GP IgG ELISA validation, accuracy was assessed via dilutional linearity by evaluating whether the assay could generate results that are proportional to the expected concentrations of anti-EBOV GP IgG in diluted test samples over a series of dilution levels. Each dilutional linearity VTS was evaluated in the assay a minimum of two times by four individual operators over two consecutive days (eight independent analyses per VTS). Concentrations and endpoint titers were calculated for each test sample run. Results were log-transformed, compared to log-transformed spike level, and a random regression model was fit to the observations. The percent total error was then calculated from the model, using a 50% cutoff as the desired maximum percent total error.

#### Limit of detection

The LOD is defined as the lowest predicted value for which there is 95% probability that an estimated value can be obtained. To determine the LOD, results obtained from the 100 VTSs generated for dilutional linearity were used in a logistic regression analysis to predict the probability that the concentration or endpoint titer could be determined as a function of its predicted log-transformed concentration or endpoint titer. The LOD was then estimated from the models as the lowest predicted concentration or endpoint titer with at least 95% probability of determination.

#### Limits of quantitation

The ULOQ and lower limit of quantitation (LLOQ) represent the concentrations or endpoint titers that bound the range of values for which dilutional linearity and precision are demonstrated. The ULOQ was calculated by fitting a random regression model relating log-transformed concentration or endpoint titer to log-transformed spike level. The percent total error for each test specimen was calculated using the regression model, and results were back-transformed to the observational scale. Using a 50% or 60% cutoff for concentration or endpoint titer, respectively, the final LLOQ was calculated as the average of the ten corresponding averages from the last dilution level with acceptable accuracy. All %CVs at the first dilution step for each ULOQ VTS were less than 40%; therefore, the ULOQ was set at the maximum mean concentration across the three test specimens.

#### Precision

The precision of an analytical method describes the closeness of agreement of individual measures of an analyte when the procedure is applied repeatedly to multiple aliquots of a single homogeneous volume of biological matrix. For the anti-EBOV GP IgG ELISA, two aspects of precision were assessed: intermediate precision, which measures the precision between runs of the assay separated temporally, and incorporates operator-to-operator, day-to-day, and plate-to-plate variation; and repeatability, which measures precision during a single analytical run performed by a single operator using one set of equipment. To assess intermediate precision and repeatability, the 100 VTSs generated for dilutional linearity were used, as well as 25 incurred (real-world) samples representing test samples typically observed during clinical testing that span the range of the assay. These additional samples were previously determined to be in ranges of 50–100, 100–250, 250–500, 500–3,000, and above 3,000 ELISA units/mL (4–6 individual samples per range).

To calculate the intermediate precision of the assay, observations with predicted ELISA concentrations or endpoint titers within the final limits of quantitation were used to generate a mixed effect ANOVA model that included random effects for operator, day, plate-to-plate, residual error, test specimen, and test specimen x log-transformed spike level. The estimated variance components from the model were then used to calculate repeatability and intermediate precision, expressed as the percent coefficient of variation (CV) across all test samples. The %CV for each source of variance was calculated as $100\;x\sqrt{e^{\ln{\left(10\right)}^{2}\;x\;\sigma^{2}}-1}$ where σ^2^ is the model-estimated variance for the specific variance source. The %CV for the intermediate precision of the assay was calculated as $100\;x\sqrt{e^{\ln{\left(10\right)}^{2}\;x\;\sigma_{T}^{2}}-1}$ where $\sigma_{T}^{2}$ is the sum of the model-estimated variances for day, operator, and plate for the same test sample and spike level. The %CV associated with the residual variance serves as an estimate for the assay repeatability with respect to concentration. Total assay variability due to the assay method was also calculated as the sum of the intermediate precision and repeatability components of the ANOVA model.

Due to the discrete nature of endpoint titer, the repeatability of the assay with respect to this value was determined by first calculating the percent of results for each VTS that were within one dilution (within two-fold) of the respective median titer of the replicates across all plates. The acceptable percentage of replicates on a plate for a given test sample where the reported endpoint titer was greater than a two-fold difference from the median was set at 10%, and at least 80% of the test samples were required to meet this criterion for assay acceptance.

#### Selectivity

The selectivity of the assay is the ability of the assay to ensure that components of the sample matrix do not interfere with quantification of anti-EBOV GP IgG antibodies. The observed concentration or endpoint titer was used to calculate a percent difference from the expected concentration or endpoint titer for each positive test sample. These values were compared to the mean difference across the five naïve human serum samples using an equivalence analysis to determine if the confidence interval (CI) was completely contained within the validation acceptance interval for concentration (-35 to 35%) or endpoint titer (0.34 to 3.00).

#### Interference

Interference is the ability of the anti-EBOV GP IgG ELISA to differentiate and quantify the EBOV GP-specific antibodies in the presence of other potential-interfering molecules typically encountered in serum. For each interferent tested (see section 2.7), the percent or fold difference of the interferent concentration or endpoint titer, respectively, and corresponding mock sample values were calculated for each of the four test samples. The mean difference across the four test samples and a corresponding 90% confidence interval on this mean were calculated. An equivalence analysis (two one-sided tests) was conducted to determine if the confidence interval was completely contained within the validation acceptance intervals (-45% to 45% for concentration, 0.34 to 3.00 for endpoint titer).

#### Specificity

Specificity is the ability of the anti-EBOV GP IgG ELISA to differentiate EBOV GP-specific antibodies from nonspecific analytes potentially present in human serum. For each of the 10 test samples, the difference of the log-transformed mock concentration or endpoint titer and log-transformed antigen-adsorbed concentration or endpoint titer was calculated. The mean difference across the test samples and corresponding lower 90% confidence bound on this mean were then calculated. The results were back-transformed to the observational scale to obtain a ratio of geometric means. An equivalence analysis (two one-sided tests) was conducted to determine if the lower confidence bound on this ratio was greater or less than the validation acceptance limit. For the 25 μg/mL rGP and 25 μg/mL CMV antigen samples, the acceptance limits for the ratio comparisons were ≥3.7 and ≤2.5, respectively.

## Supporting information

S1 Fig Optical density (OD) values for rGP coating concentration optimization. (DOCX)

S2 Fig Optical density (OD) values and binding ratios for antibody conjugate dilution optimization. (DOCX)

S3 Fig RS curves for RS starting dilution optimization. (DOCX)

S4 Fig RS curves for RS dilution scheme optimization. (DOCX)

S5 Fig Optical density (OD) values for candidate NC serum lots. (DOCX)

S6 Fig Outlier analysis for qualification results using studentized residuals about the logarithmic (Base 10) means within QTSs versus logarithmic means. Horizontal reference lines at -3 and 3 provide boundaries for potential outliers.
(DOCX)

S7 Fig Plot relating the probability of estimating a non-zero ELISA concentration by the model-predicted ELISA concentration for determination of LOD. The LOD and upper and lower 95% confidence bounds are shown as vertical lines.
(DOCX)

S8 Fig Random straight-line regression model fit relating log10 ELISA concentration to final dilution within parent qualification test samples. Dashed lines correspond to individual test samples and solid red line is the average across test samples.
(DOCX)

S9 Fig Standardized residuals against fitted values for evaluation of parallelism between second-generation RS BMIZAIRE102 and serum BMIZAIRE116 generated from the WHO Reference Reagent 15/220. (DOCX)

S10 Fig Standardized residuals against fitted values for evaluation of parallelism between human RS and human test samples. (DOCX)

S1 Table OD values determined for the candidate PC serum at different starting dilutions. (DOCX)

S2 Table ELISA concentrations determined for the candidate QC-High and QC-Low serum controls. (DOCX)

S3 Table ELISA concentration of each qualification test sample. (XLSX)

S4 Table OD values and average values generated for 150 naïve human serum samples. (DOCX)

S5 Table Percent relative error from random regression model for each parent qualification test sample and dilution level used to determine dilutional linearity. (DOCX)

S6 Table Human serum proficiency panel members for robustness testing. (DOCX)

S7 Table Estimated geometric mean ELISA concentrations for human serum proficiency panel members when evaluated using rGP-coated plates stored at 2–8°C for up to seven days. (DOCX)

S8 Table Estimated geometric mean ELISA concentrations for the QC-High and QC-Low serum following storage at 2–8°C for up to 21 days, storage at room temperature for 24 hours, or being subjected to up to seven freeze/thaw cycles. (DOCX)

S9 Table Estimated geometric mean ELISA concentrations for human serum proficiency panel members when evaluated using rGP stored at 2–8°C for up to seven days or being subjected to up to eight freeze/thaw cycles. (DOCX)

S10 Table ELISA concentration of each validation test sample. (XLSX)

S11 Table Parent test samples, dilution factors, and starting dilutions for qualification test samples. (DOCX)

S12 Table Parent test samples, dilution factors, and starting dilutions for validation test samples. (DOCX)
