# Supplementary figures and images for: Development, qualification, and validation of the Filovirus Animal Nonclinical Group anti-Ebola virus glycoprotein immunoglobulin G enzyme-linked immunosorbent assay for human serum samples
Source: PLoS One. 2019 Apr 18;14(4):e0215457. doi: 10.1371/journal.pone.0215457 (PMC6472792; doi:10.1371/journal.pone.0215457)

**S4 Fig. RS curves for RS dilution scheme optimization.**


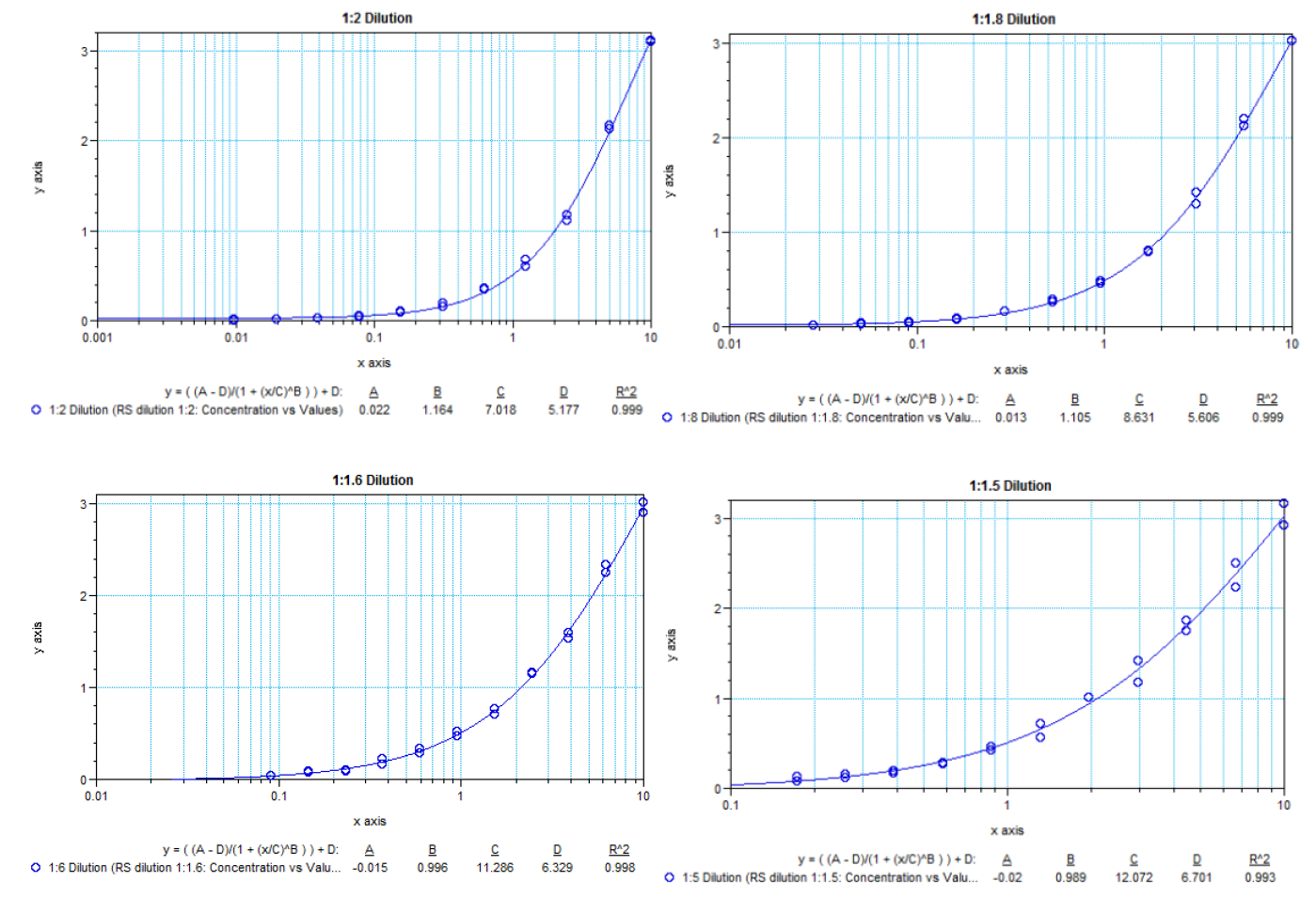

Supplement: S4 Fig — (DOCX) [file pone.0215457.s004.docx]

**S5 Fig. Optical density (OD) values for candidate NC serum lots.**


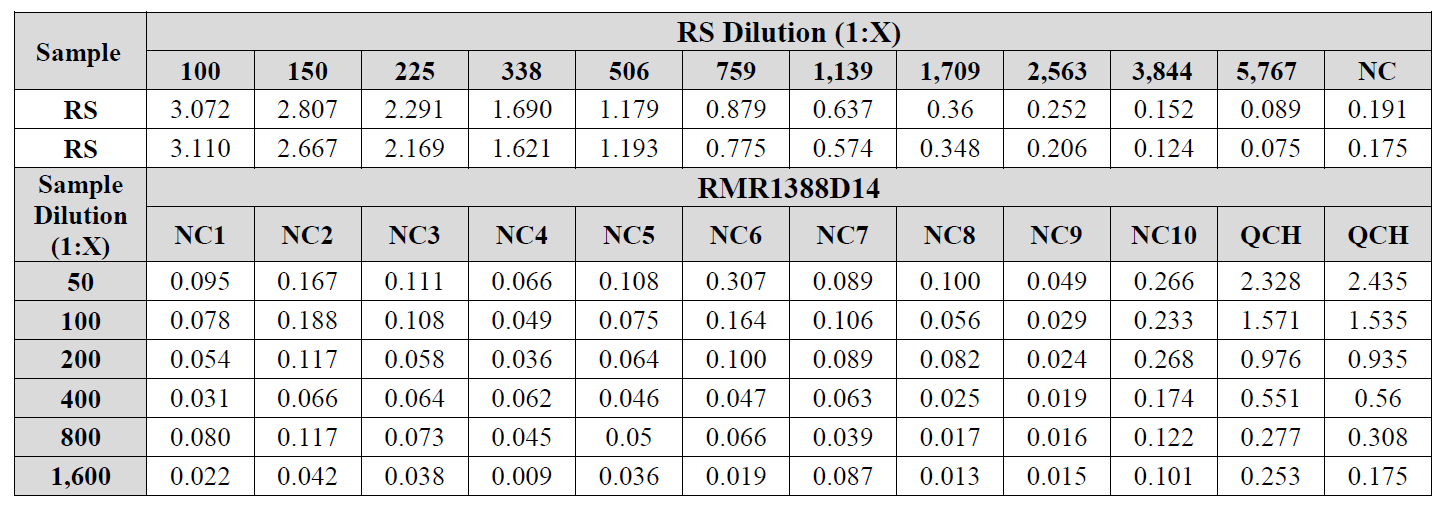

Supplement: S5 Fig — (DOCX) [file pone.0215457.s005.docx]
