# Supplementary material for: Development, qualification, and validation of the Filovirus Animal Nonclinical Group anti-Ebola virus glycoprotein immunoglobulin G enzyme-linked immunosorbent assay for human serum samples
Source: PLoS One. 2019 Apr 18;14(4):e0215457. doi: 10.1371/journal.pone.0215457 (PMC6472792; doi:10.1371/journal.pone.0215457)
Supplement: S6 Fig — Horizontal reference lines at -3 and 3 provide boundaries for potential outliers. (DOCX) [file pone.0215457.s006.docx]

**S6 Fig. Outlier analysis for qualification results using studentized residuals about the logarithmic (Base 10) means within QTSs versus logarithmic means.** Horizontal reference lines at -3 and 3 provide boundaries for potential outliers.
