# Supplementary material for: Development, qualification, and validation of the Filovirus Animal Nonclinical Group anti-Ebola virus glycoprotein immunoglobulin G enzyme-linked immunosorbent assay for human serum samples
Source: PLoS One. 2019 Apr 18;14(4):e0215457. doi: 10.1371/journal.pone.0215457 (PMC6472792; doi:10.1371/journal.pone.0215457)

**S7 Fig. Plot relating the probability of estimating a non-zero ELISA concentration by the model-predicted ELISA concentration for determination of LOD.** The LOD and upper and lower 95% confidence bounds are shown as vertical lines.


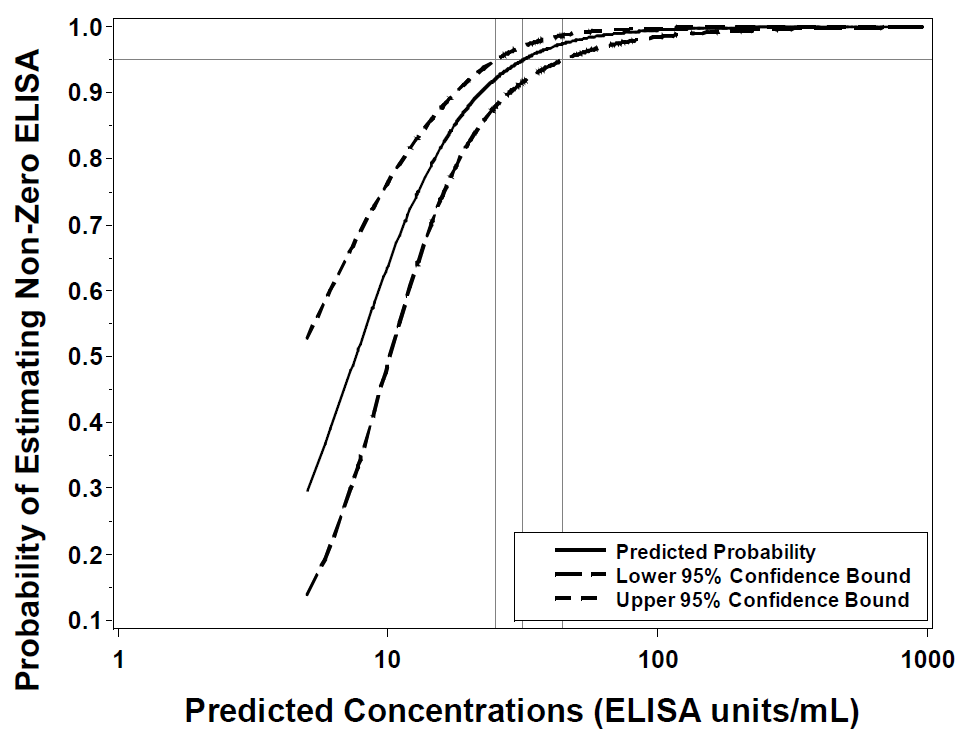

Supplement: S7 Fig — The LOD and upper and lower 95% confidence bounds are shown as vertical lines. (DOCX) [file pone.0215457.s007.docx]
