# Supplementary material for: Development, qualification, and validation of the Filovirus Animal Nonclinical Group anti-Ebola virus glycoprotein immunoglobulin G enzyme-linked immunosorbent assay for human serum samples
Source: PLoS One. 2019 Apr 18;14(4):e0215457. doi: 10.1371/journal.pone.0215457 (PMC6472792; doi:10.1371/journal.pone.0215457)
Supplement: S8 Fig — Dashed lines correspond to individual test samples and solid red line is the average across test samples. (DOCX) [file pone.0215457.s008.docx]

**S8 Fig. Random straight-line regression model fit relating log10 ELISA concentration to final dilution within parent qualification test samples.** Dashed lines correspond to individual test samples and solid red line is the average across test samples.
