# Supplementary material for: Development, qualification, and validation of the Filovirus Animal Nonclinical Group anti-Ebola virus glycoprotein immunoglobulin G enzyme-linked immunosorbent assay for human serum samples
Source: PLoS One. 2019 Apr 18;14(4):e0215457. doi: 10.1371/journal.pone.0215457 (PMC6472792; doi:10.1371/journal.pone.0215457)
Supplement: S9 Fig — (DOCX) [file pone.0215457.s009.docx]

**S9 Fig. Standardized residuals against fitted values for evaluation of parallelism between second-generation RS BMIZAIRE102 and serum BMIZAIRE116 generated from the WHO reference reagent 15/220.**
