# Supplementary material for: Development, qualification, and validation of the Filovirus Animal Nonclinical Group anti-Ebola virus glycoprotein immunoglobulin G enzyme-linked immunosorbent assay for human serum samples
Source: PLoS One. 2019 Apr 18;14(4):e0215457. doi: 10.1371/journal.pone.0215457 (PMC6472792; doi:10.1371/journal.pone.0215457)
Supplement: S1 Table — (DOCX) [file pone.0215457.s011.docx]

**S1 Table. OD values determined for the candidate PC serum at different starting dilutions.**

|  | **Serum Starting Dilution**  **(1:X)** | **Serum Dilution (1:X)** | | | | | | | | | | | |
| --- | --- | --- | --- | --- | --- | --- | --- | --- | --- | --- | --- | --- | --- |
|  |  |  |  |  |  |  |  |  |  |  |  |  | **Blank** |
|  |  | **1** | **2** | **3** | **4** | **5** | **6** | **7** | **8** | **9** | **10** | **11** | **12** |
|  | **Positive Serum (RMR1388D31)** | | | | | | | | | | | | |
| **A** | **50** | 3.660 | 3.037 | 1.867 | 1.070 | 0.541 | 0.274 | 0.157 | 0.058 | 0.032 | 0.012 | 0.017 | 0.000 |
| **B** | **80** | 3.322 | 2.243 | 1.193 | 0.664 | 0.356 | 0.163 | 0.096 | 0.038 | 0.021 | 0.006 | 0.002 | 0.000 |
| **C** | **100** | 2.913 | 1.725 | 0.954 | 0.514 | 0.252 | 0.142 | 0.072 | 0.031 | 0.014 | 0.004 | 0.001 | 0.001 |
| **D** | **200** | 1.693 | 0.936 | 0.507 | 0.244 | 0.119 | 0.059 | 0.032 | 0.013 | 0.003 | 0.000 | 0.000 | -0.001 |
|  |  | **NC1** | | **NC2** | | **NC3** | | **NC4** | | -- | -- | -- | -- |
| **E** | **50** | 0.139 | 0.133 | 0.120 | 0.143 | 0.192 | 0.225 | 0.284 | 0.345 | -- | -- | -- | -- |
| **F** | -- | -- | -- | -- | -- | -- | -- | -- | -- | -- | -- | -- | -- |
| **G** | -- | -- | -- | -- | -- | -- | -- | -- | -- | -- | -- | -- | -- |
| **H** | -- | -- | -- | -- | -- | -- | -- | -- | -- | -- | -- | -- | -- |
