# Supplementary material for: Development, qualification, and validation of the Filovirus Animal Nonclinical Group anti-Ebola virus glycoprotein immunoglobulin G enzyme-linked immunosorbent assay for human serum samples
Source: PLoS One. 2019 Apr 18;14(4):e0215457. doi: 10.1371/journal.pone.0215457 (PMC6472792; doi:10.1371/journal.pone.0215457)
Supplement: S2 Table — (DOCX) [file pone.0215457.s012.docx]

**S2 Table. ELISA concentrations determined for the candidate QC-High and QC-Low serum controls.**

|  | **QC-High**  **(ELISA units/mL)** | **QC-Low**  **(ELISA units/mL)** |
| --- | --- | --- |
|  | 488.31 | 139.88 |
|  | 416.67 | 105.86 |
|  | 452.68 | 99.02 |
|  | 478.50 | 160.77 |
|  | 428.45 | 105.92 |
|  | 447.55 | 93.45 |
| Average | 452.02 | 117.49 |
| Standard Deviation | 27.73 | 26.69 |
| %CV | 6.13% | 22.72% |
