# Supplementary material for: Development, qualification, and validation of the Filovirus Animal Nonclinical Group anti-Ebola virus glycoprotein immunoglobulin G enzyme-linked immunosorbent assay for human serum samples
Source: PLoS One. 2019 Apr 18;14(4):e0215457. doi: 10.1371/journal.pone.0215457 (PMC6472792; doi:10.1371/journal.pone.0215457)
Supplement: S4 Table — (DOCX) [file pone.0215457.s014.docx]

**S4 Table. OD values and average values for 150 naïve human serum samples.**

| **Test Sample #** | **OD1** | **OD2** | **Average OD** |
| --- | --- | --- | --- |
| 1 | 0.302 | 0.318 | 0.310 |
| 2 | 0.288 | 0.193 | 0.240 |
| 3 | 0.216 | 0.139 | 0.177 |
| 4 | 0.131 | 0.159 | 0.145 |
| 5 | 0.220 | 0.545 | 0.382 |
| 6 | 0.159 | 0.121 | 0.140 |
| 7 | 0.106 | 0.104 | 0.105 |
| 8 | 0.221 | 0.155 | 0.188 |
| 9 | 0.111 | 0.131 | 0.121 |
| 10 | 0.075 | 0.085 | 0.080 |
| 11 | 0.155 | 0.161 | 0.158 |
| 12 | 0.208 | 0.313 | 0.260 |
| 13 | 0.176 | 0.237 | 0.206 |
| 14 | 0.197 | 0.237 | 0.217 |
| 15 | 0.130 | 0.173 | 0.152 |
| 16 | 0.126 | 0.146 | 0.136 |
| 17 | 0.110 | 0.149 | 0.129 |
| 18 | 0.134 | 0.199 | 0.166 |
| 19 | 0.115 | 0.115 | 0.115 |
| 20 | 0.109 | 0.102 | 0.105 |
| 21 | 0.202 | 0.178 | 0.190 |
| 22 | 0.221 | 0.242 | 0.231 |
| 23 | 0.078 | 0.140 | 0.109 |
| 24 | 0.105 | 0.130 | 0.117 |
| 25 | 0.079 | 0.075 | 0.077 |
| 26 | 0.107 | 0.074 | 0.090 |
| 27 | 0.147 | 0.158 | 0.152 |
| 28 | 0.169 | 0.154 | 0.161 |
| 29 | 0.203 | 0.293 | 0.248 |
| 30 | 0.137 | 0.149 | 0.143 |
| 31 | 0.142 | 0.138 | 0.140 |
| 32 | 0.070 | 0.068 | 0.069 |
| 33 | 0.175 | 0.182 | 0.178 |
| 34 | 0.159 | 0.157 | 0.158 |
| 35 | 0.135 | 0.168 | 0.151 |
| 36 | 0.183 | 0.169 | 0.176 |
| 37 | 0.130 | 0.119 | 0.124 |
| 38 | 0.102 | 0.104 | 0.103 |
| 39 | 0.124 | 0.141 | 0.133 |
| 40 | 0.104 | 0.125 | 0.115 |
| 41 | 0.097 | 0.092 | 0.095 |
| 42 | 0.125 | 0.144 | 0.135 |
| 43 | 0.094 | 0.092 | 0.093 |
| 44 | 0.081 | 0.103 | 0.092 |
| 45 | 0.169 | 0.104 | 0.137 |
| 46 | 0.123 | 0.126 | 0.124 |
| 47 | 0.143 | 0.164 | 0.153 |
| 48 | 0.059 | 0.232 | 0.145 |
| 49 | 0.067 | 0.078 | 0.073 |
| 50 | 0.092 | 0.101 | 0.097 |
| 51 | 0.076 | 0.086 | 0.081 |
| 52 | 0.117 | 0.113 | 0.115 |
| 53 | 0.192 | 0.348 | 0.270 |
| 54 | 0.162 | 0.170 | 0.166 |
| 55 | 0.106 | 0.112 | 0.109 |
| 56 | 0.132 | 0.147 | 0.139 |
| 57 | 0.081 | 0.072 | 0.076 |
| 58 | 0.091 | 0.080 | 0.085 |
| 59 | 0.086 | 0.144 | 0.115 |
| 60 | 0.069 | 0.091 | 0.080 |
| 61 | 0.207 | 0.196 | 0.202 |
| 62 | 0.857 | 0.842 | 0.850 |
| 63 | 0.258 | 0.275 | 0.267 |
| 64 | 0.107 | 0.102 | 0.104 |
| 65 | 0.222 | 0.263 | 0.242 |
| 66 | 0.077 | 0.100 | 0.088 |
| 67 | 0.097 | 0.090 | 0.094 |
| 68 | 0.093 | 0.073 | 0.083 |
| 69 | 0.100 | 0.105 | 0.103 |
| 70 | 0.079 | 0.078 | 0.079 |
| 71 | 0.093 | 0.105 | 0.099 |
| 72 | 0.138 | 0.123 | 0.130 |
| 73 | 0.058 | 0.062 | 0.060 |
| 74 | 0.097 | 0.108 | 0.103 |
| 75 | 0.133 | 0.100 | 0.116 |
| 76 | 0.108 | 0.103 | 0.106 |
| 77 | 0.068 | 0.083 | 0.075 |
| 78 | 0.116 | 0.135 | 0.126 |
| 79 | 0.064 | 0.075 | 0.069 |
| 80 | 0.069 | 0.078 | 0.073 |
| 81 | 0.095 | 0.100 | 0.097 |
| 82 | 0.082 | 0.089 | 0.085 |
| 83 | 0.095 | 0.091 | 0.093 |
| 84 | 0.116 | 0.109 | 0.112 |
| 85 | 0.156 | 0.158 | 0.157 |
| 86 | 0.101 | 0.109 | 0.105 |
| 87 | 0.279 | 0.314 | 0.297 |
| 88 | 0.095 | 0.092 | 0.093 |
| 89 | 0.111 | 0.156 | 0.133 |
| 90 | 0.063 | 0.075 | 0.069 |
| 91 | 0.108 | 0.156 | 0.132 |
| 92 | 0.121 | 0.093 | 0.107 |
| 93 | 0.067 | 0.184 | 0.126 |
| 94 | 0.103 | 0.052 | 0.077 |
| 95 | 0.150 | 0.060 | 0.105 |
| 96 | 0.088 | 0.068 | 0.078 |
| 97 | 0.090 | 0.095 | 0.092 |
| 98 | 0.088 | 0.163 | 0.126 |
| 99 | 3.748 | 3.628 | 3.688 |
| 100 | 3.736 | 3.006 | 3.371 |
| 101 | 0.127 | 0.129 | 0.128 |
| 102 | 0.213 | 0.220 | 0.217 |
| 103 | 0.225 | 0.228 | 0.227 |
| 104 | 0.126 | 0.103 | 0.115 |
| 105 | 0.072 | 0.069 | 0.071 |
| 106 | 0.059 | 0.147 | 0.103 |
| 107 | 0.176 | 0.153 | 0.165 |
| 108 | 0.248 | 0.192 | 0.220 |
| 109 | 0.159 | 0.181 | 0.170 |
| 110 | 0.126 | 0.114 | 0.120 |
| 111 | 0.228 | 0.185 | 0.207 |
| 112 | 0.040 | 0.027 | 0.034 |
| 113 | 0.155 | 0.154 | 0.155 |
| 114 | 0.135 | 0.129 | 0.132 |
| 115 | 0.113 | 0.124 | 0.119 |
| 116 | 0.785 | 0.673 | 0.729 |
| 117 | 0.139 | 0.168 | 0.154 |
| 118 | 0.079 | 0.057 | 0.068 |
| 119 | 0.188 | 0.092 | 0.140 |
| 120 | 0.099 | 0.092 | 0.096 |
| 121 | 0.118 | 0.136 | 0.127 |
| 122 | 0.055 | 0.050 | 0.053 |
| 123 | 0.129 | 0.120 | 0.125 |
| 124 | 0.116 | 0.122 | 0.119 |
| 125 | 0.151 | 0.111 | 0.131 |
| 126 | 0.083 | 0.110 | 0.097 |
| 127 | 0.129 | 0.214 | 0.172 |
| 128 | 0.155 | 0.233 | 0.194 |
| 129 | 0.087 | 0.065 | 0.076 |
| 130 | 0.135 | 0.153 | 0.144 |
| 131 | 0.120 | 0.133 | 0.127 |
| 132 | 0.174 | 0.176 | 0.175 |
| 133 | 0.116 | 0.128 | 0.122 |
| 134 | 0.121 | 0.093 | 0.107 |
| 135 | 0.035 | 0.077 | 0.056 |
| 136 | 0.070 | 0.023 | 0.047 |
| 137 | 0.053 | 0.049 | 0.051 |
| 138 | 0.165 | 0.069 | 0.117 |
| 139 | 0.303 | 0.329 | 0.316 |
| 140 | 0.119 | 0.282 | 0.201 |
| 141 | 0.135 | 0.107 | 0.121 |
| 142 | 0.093 | 0.122 | 0.108 |
| 143 | 0.042 | 0.020 | 0.031 |
| 144 | 0.114 | 0.099 | 0.107 |
| 145 | 0.072 | 0.059 | 0.066 |
| 146 | 0.100 | 0.075 | 0.088 |
| 147 | 0.200 | 0.187 | 0.194 |
| 148 | 0.153 | 0.145 | 0.149 |
| 149 | 0.035 | 0.073 | 0.054 |
| 150 | 0.064 | 0.049 | 0.057 |
