# Supplementary material for: Development, qualification, and validation of the Filovirus Animal Nonclinical Group anti-Ebola virus glycoprotein immunoglobulin G enzyme-linked immunosorbent assay for human serum samples
Source: PLoS One. 2019 Apr 18;14(4):e0215457. doi: 10.1371/journal.pone.0215457 (PMC6472792; doi:10.1371/journal.pone.0215457)
Supplement: S5 Table — (DOCX) [file pone.0215457.s015.docx]

**S5 Table. Percent relative error from random regression model for each parent qualification test sample and dilution level used to determine dilutional linearity.**

| **Parent Test Sample** | **Dilution Level** | **Percent Relative Error** |
| --- | --- | --- |
| BMIZAIRE105 | 3 | 14.81 |
|  | 4 | 26.49 |
|  | 5 | 24.36 |
|  | 6 | 24.20 |
|  | 7 | 47.60 |
|  | 8 | 92.39 |
|  | 9 | >100 |
|  | 10 | >100 |
| 2163-005, Day 14 | 3 | 17.81 |
|  | 4 | 16.93 |
|  | 5 | 12.87 |
|  | 6 | 9.63 |
|  | 7 | 20.44 |
|  | 8 | 28.00 |
|  | 9 | 16.88 |
|  | 10 | 29.79 |
| 2163-006, Day 14 | 3 | 21.67 |
|  | 4 | 20.88 |
|  | 5 | 24.09 |
|  | 6 | >100 |
|  | 7 | 65.40 |
|  | 8 | >100 |
|  | 9 | >100 |
|  | 10 | >100 |
| 2163-010, Day 14 | 3 | 15.06 |
|  | 4 | 29.41 |
|  | 5 | 18.02 |
|  | 6 | >100 |
|  | 7 | >100 |
|  | 8 | >100 |
|  | 9 | >100 |
|  | 10 | >100 |
| 2163-013, Day 180 | 3 | 15.36 |
|  | 4 | 17.61 |
|  | 5 | 37.07 |
|  | 6 | 38.04 |
|  | 7 | >100 |
|  | 8 | >100 |
|  | 9 | >100 |
|  | 10 | >100 |
| 2163-016, Day 180 | 3 | 30.80 |
|  | 4 | 26.77 |
|  | 5 | 29.55 |
|  | 6 | >100 |
|  | 7 | 55.90 |
|  | 8 | >100 |
|  | 9 | >100 |
|  | 10 | >100 |
| 2163-023, Day 84 | 3 | 25.22 |
|  | 4 | 17.32 |
|  | 5 | 34.10 |
|  | 6 | >100 |
|  | 7 | >100 |
|  | 8 | >100 |
|  | 9 | >100 |
|  | 10 | >100 |
| 2163-024, Day 84 | 3 | 16.97 |
|  | 4 | 49.43 |
|  | 5 | 32.56 |
|  | 6 | >100 |
|  | 7 | >100 |
|  | 8 | >100 |
|  | 9 | >100 |
|  | 10 | >100 |
| 2163-034, Day 84 | 3 | 17.18 |
|  | 4 | 5.72 |
|  | 5 | 13.94 |
|  | 6 | 13.20 |
|  | 7 | 28.89 |
|  | 8 | 53.45 |
|  | 9 | 41.16 |
|  | 10 | >100 |
| 2163-071, Day 28 | 3 | 11.87 |
|  | 4 | 8.08 |
|  | 5 | 19.36 |
|  | 6 | 24.89 |
|  | 7 | 36.65 |
|  | 8 | 50.14 |
|  | 9 | >100 |
|  | 10 | >100 |
