# Supplementary material for: Development, qualification, and validation of the Filovirus Animal Nonclinical Group anti-Ebola virus glycoprotein immunoglobulin G enzyme-linked immunosorbent assay for human serum samples
Source: PLoS One. 2019 Apr 18;14(4):e0215457. doi: 10.1371/journal.pone.0215457 (PMC6472792; doi:10.1371/journal.pone.0215457)
Supplement: S6 Table — (DOCX) [file pone.0215457.s016.docx]

**S6 Table. Human serum proficiency panel members for robustness testing.**

| **Sample Identity** | **Volume BMI529 (Naïve Diluent in mL)** | **Volume of BMIZAIRE105.a (mL)** | **Approximate Concentration (ELISA Units/mL)** |
| --- | --- | --- | --- |
| BMIZAIRE105.a | N/A | N/A | 1031 |
| BMI-ZPP-15 | 2.20 | 7.80 | 800 |
| BMI-ZPP-14 | 3.20 | 6.80 | 700 |
| BMI-ZPP-11 | 4.20 | 5.80 | 600 |
| BMI-ZPP-16 | 5.20 | 4.80 | 500 |
| BMI-ZPP-12 | 6.10 | 3.90 | 400 |
| BMI-ZPP-13 | 7.10 | 2.90 | 300 |
| BMI-ZPP-17 | 8.10 | 1.90 | 200 |
| BMI-ZPP-19 | 9.00 | 1.00 | 100 |
| BMI-ZPP-20 | 9.50 | 0.50 | 50 |
| BMI-ZPP-18 | 10.00 | 0.00 | 0 |
