# Supplementary material for: Development, qualification, and validation of the Filovirus Animal Nonclinical Group anti-Ebola virus glycoprotein immunoglobulin G enzyme-linked immunosorbent assay for human serum samples
Source: PLoS One. 2019 Apr 18;14(4):e0215457. doi: 10.1371/journal.pone.0215457 (PMC6472792; doi:10.1371/journal.pone.0215457)
Supplement: S7 Table — (DOCX) [file pone.0215457.s017.docx]

Table S7. Estimated geometric mean ELISA concentrations for human serum proficiency panel members when evaluated using rGP-coated plates stored at 2-8°C for up to seven days.

| **Sample ID** | **Estimated Geometric Mean Concentration (Two-Sided 95% Confidence Bounds)** | | | | |
| --- | --- | --- | --- | --- | --- |
|  | **Day 1** | **Lower and Upper Acceptance Criteria (70% and 130% of Day 1 Mean)** | **Day 3** | **Day 5** | **Day 7** |
| BMI-ZPP-11 | 501.23 (444.13, 565.67) | 350.86, 651.60 | 522.68 (466.53, 585.59) | 545.06 (484.80, 612.80) | 568.39 (498.80, 647.69) |
| BMI-ZPP-12 | 324.48 (287.52, 366.20) | 227.14, 421.82 | 334.43 (298.50, 374.68) | 344.68 (306.58, 387.52) | 355.25 (311.75, 404.81) |
| BMI-ZPP-13 | 238.84 (211.64, 269.55) | 167.19, 310.49 | 244.14 (217.92, 273.53) | 249.56 (221.97, 280.58) | 255.10 (223.86, 290.69) |
| BMI-ZPP-14 | 529.70 (469.35, 597.80) | 370.79, 688.61 | 553.19 (493.77, 619.77) | 577.73 (513.86, 649.54) | 603.36 (529.49, 687.54) |
| BMI-ZPP-15 | 645.75 (572.18, 728.77) | 452.03, 839.48 | 678.00 (605.17, 759.60) | 711.87 (633.17, 800.35) | 747.42 (655.91, 851.70)* |
| BMI-ZPP-16 | 386.03 (340.91, 437.13) | 270.22, 501.84 | 399.73 (355.56, 449.39) | 413.92 (366.97, 466.88) | 428.61 (375.08, 489.78) |
| BMI-ZPP-17 | 161.68 (143.26, 182.47) | 113.18, 210.18 | 163.54 (145.97, 183.22) | 165.42 (147.13, 185.98) | 167.32 (146.84, 190.67) |
| BMI-ZPP-19 | 82.19 (72.82, 92.75) | 57.53, 106.85 | 81.63 (72.86, 91.45) | 81.08 (72.11, 91.15) | 80.53 (70.67, 91.76) |

(*) The upper confidence bound was greater than the upper acceptance criteria value for that coating day
