# Supplementary material for: Development, qualification, and validation of the Filovirus Animal Nonclinical Group anti-Ebola virus glycoprotein immunoglobulin G enzyme-linked immunosorbent assay for human serum samples
Source: PLoS One. 2019 Apr 18;14(4):e0215457. doi: 10.1371/journal.pone.0215457 (PMC6472792; doi:10.1371/journal.pone.0215457)
Supplement: S8 Table — (DOCX) [file pone.0215457.s018.docx]

Table S8. Estimated geometric mean ELISA concentrations for the QC-High and QC-Low serum following storage at 2-8°C for up to 21 days, storage at room temperature for 24 hours, or being subjected to up to seven freeze/thaw cycles.

| **Results for Serum Storage at 2-8°C or Room Temperature** | | | | | | | | | | | |
| --- | --- | --- | --- | --- | --- | --- | --- | --- | --- | --- | --- |
| **Serum** | **Estimated Geometric Mean Concentration (Two-Sided 95% Confidence Bounds)** | | | | | | | | | | |
|  | **Day 0 at 2-8°C**  **(Baseline)** | **Lower and Upper Acceptance Criteria (70% and 130% of Day 0 Mean)** | | **1 Day at 2-8°C** | | **24 Hours at Room Temperature** | **7 Days at 2-8°C** | | **14 Days at 2-8°C** | | **21 Days at 2-8°C** |
| BMIZAIRE103 | 655.16  (622.30, 689.76) | 458.61, 851.71 | | 657.26  (625.56, 690.58) | | 657.47  (625.87, 690.67) | 669.99  (641.59, 699.65) | | 685.16  (650.92, 721.19) | | 700.66  (653.30, 751.45) |
| BMIZAIRE104 | 163.18  (155.0, 171.80) | 114.23, 212.13 | | 162.53  (154.69, 170.77) | | 162.46  (154.66, 170.67) | 158.68  (151.95, 165.71) | | 154.31  (146.60, 162.42) | | 150.05  (139.91, 160.93) |
|  | | | | | | | | | | | |
| **Results for Serum Freeze/Thaw Cycles** | | | | | | | | | | | |
| **Serum** | **Estimated Geometric Mean Concentration (Two-Sided 95% Confidence Bounds)** | | | | | | | | | | |
|  | **1 Cycle (Baseline)** | | **Lower and Upper Acceptance Criteria (70% and 130% of Day 0 Mean)** | | **3 Cycles** | | | **5 Cycles** | | **7 Cycles** | |
| BMIZAIRE103 | 610.73  (529.83, 703.97) | | 427.51, 793.95 | | 585.52  (526.82, 650.77) | | | 561.36  (507.41, 621.04) | | 538.19  (471.81, 613.92) | |
| BMIZAIRE104 | 148.36  (128.71, 171.01) | | 103.85, 192.87 | | 150.85  (135.72, 167.66) | | | 153.38  (138.64, 169.69) | | 155.95  (136.72, 177.90) | |
