# Supplementary material for: Development, qualification, and validation of the Filovirus Animal Nonclinical Group anti-Ebola virus glycoprotein immunoglobulin G enzyme-linked immunosorbent assay for human serum samples
Source: PLoS One. 2019 Apr 18;14(4):e0215457. doi: 10.1371/journal.pone.0215457 (PMC6472792; doi:10.1371/journal.pone.0215457)
Supplement: S9 Table — (DOCX) [file pone.0215457.s019.docx]

Table S9. Estimated geometric mean ELISA concentrations for human serum proficiency panel members when evaluated using rGP stored at 2-8°C for up to seven days or being subjected to up to eight freeze/thaw cycles.

| **Results following rGP storage at 2-8°C for up to seven days** | | | | | |
| --- | --- | --- | --- | --- | --- |
| **Sample ID** | **Estimated Geometric Mean Concentration (Two-Sided 95% Confidence Bounds)** | | | | |
|  | **Day 1 (Baseline)** | **Lower and Upper Acceptance Criteria (70% and 130% of Day 1 Mean)** | **Day 3** | **Day 5** | **Day 7** |
| BMI-ZPP-11 | 572.90 (493.87, 664.58) | 401.03, 744.77 | 636.84 (558.08, 726.71) | 683.39 (598.37, 780.48)* | 733.33 (635.50, 846.23)* |
| BMI-ZPP-12 | 327.20 (282.83, 378.54) | 229.04, 425.36 | 372.83 (328.45, 423.21) | 406.73 (358.49, 461.46)* | 443.71 (387.33, 508.30)* |
| BMI-ZPP-13 | 219.87 (188.88, 255.96) | 153.91, 285.83 | 254.97 (223.22, 291.24)* | 281.43 (246.64, 321.13)* | 310.63 (269.85, 357.58)* |
| BMI-ZPP-14 | 581.06 (496.53, 679.98) | 406.74, 755.38 | 645.51 (561.39, 742.23) | 692.40 (602.52, 795.69)* | 742.70 (640.69, 860.94)* |
| BMI-ZPP-15 | 730.46 (631.39, 845.06) | 511.32, 949.60 | 803.32 (707.69, 911.87) | 855.89 (754.38, 971.05)* | 911.89 (796.02, 1044.6)* |
| BMI-ZPP-16 | 435.47 (376.41, 503.79) | 304.83, 566.11 | 489.97 (431.64, 556.18) | 530.04 (467.18, 601.36)* | 573.39 (500.53, 656.86)* |
| BMI-ZPP-17 | 150.36 (129.97, 173.95) | 105.25, 195.47 | 177.31 (156.20, 201.27)* | 197.91 (174.44, 224.54)* | 220.90 (192.83, 253.06)* |
| BMI-ZPP-19 | 63.04 (54.49, 72.93) | 44.13, 81.95 | 77.25 (68.05, 87.68)* | 88.46 (77.96, 100.36)* | 101.29 (88.42, 116.04)* |
|  | | | | | |
| **Results following rGP freeze/thaw for up to eight cycles** | | | | | |
| **Sample ID** | **Estimated Geometric Mean Concentration (Two-Sided 95% Confidence Bounds)** | | | | |
|  | **2 Cycles (Baseline)** | **Lower and Upper Acceptance Criteria (70% and 130% of Day 1 Mean)** | **4 Cycles** | **6 Cycles** | **8 Cycles** |
| BMI-ZPP-11 | 633.28 (589.51, 680.29) | 443.30, 823.26 | 660.36 (618.55, 705.00) | 688.61 (645.05, 735.11) | 718.06 (668.57, 771.21) |
| BMI-ZPP-12 | 389.78 (362.85, 418.72) | 272.85, 506.71 | 406.70 (380.95, 434.20) | 424.36 (397.52, 453.02) | 442.79 (412.27, 475.56) |
| BMI-ZPP-13 | 293.53 (273.25, 315.32) | 205.47, 381.59 | 306.38 (286.98, 327.10) | 319.80 (299.57, 341.40) | 333.81 (310.80, 358.52) |
| BMI-ZPP-14 | 731.24 (680.71, 785.52) | 511.87, 950.61 | 762.37 (714.10, 813.91) | 794.84 (744.56, 848.51) | 828.68 (771.57, 890.02) |
| BMI-ZPP-15 | 825.69 (768.64, 886.99) | 577.98, 1073.4 | 860.72 (806.22, 918.91) | 897.23 (840.47, 957.82) | 935.29 (870.83, 1004.5) |
| BMI-ZPP-16 | 476.84 (443.89, 512.24) | 333.79, 619.89 | 497.41 (465.92, 531.04) | 518.88 (486.05, 553.91) | 541.26 (503.96, 581.33) |
| BMI-ZPP-17 | 183.04 (170.39, 196.63) | 128.13, 237.95 | 191.17 (179.07, 204.10) | 199.66 (187.03, 213.15) | 208.53 (194.16, 223.97) |
| BMI-ZPP-19^#^ | 92.35 (85.49, 99.77) | 64.65, 120.06 | 96.54 (89.88, 103.70) | 100.92 (93.96, 108.39) | 105.49 (97.67, 113.93) |

(*) The upper confidence bound was greater than the upper acceptance criteria value for that coating day

(#) One low ELISA value of zero observed at four cycles was removed prior to fitting the model.
