# Supplementary material for: Development, qualification, and validation of the Filovirus Animal Nonclinical Group anti-Ebola virus glycoprotein immunoglobulin G enzyme-linked immunosorbent assay for human serum samples
Source: PLoS One. 2019 Apr 18;14(4):e0215457. doi: 10.1371/journal.pone.0215457 (PMC6472792; doi:10.1371/journal.pone.0215457)
Supplement: S11 Table — (DOCX) [file pone.0215457.s021.docx]

**S11 Table. Parent test samples, dilution factors, and starting dilutions for qualification test samples.**

| **QTS** | **Volume TS (µL)** | **Volume NC (µL)** | **Starting Dilution** | **Fold-Dilution** | **Final Dilution** |
| --- | --- | --- | --- | --- | --- |
| **Parent TS: BMIZAIRE105** | | | | | |
| TS01-01 | 400.0 | 0.0 | 1 | 1.00 | 1 |
| TS01-02 | 100.0 | 100.0 | 1 | 2.00 | 2 |
| TS01-03 | 135.0 | 405.0 | 1 | 4.00 | 4 |
| TS01-04 | 35.0 | 175.0 | 1 | 6.00 | 6 |
| TS01-05 | 50.0 | 350.0 | 1 | 8.00 | 8 |
| TS01-06 | 100.0 | 300.0 | 4 | 4.00 | 16 |
| TS01-07 | 25.0 | 175.0 | 4 | 8.00 | 32 |
| TS01-08 | 75.0 | 150.0 | 16 | 3.00 | 48 |
| TS01-09 | 45.0 | 157.5 | 16 | 4.50 | 72 |
| TS01-10 | 35.0 | 201.3 | 16 | 6.75 | 108 |
| **Parent TS: 2163-005, Day 14** | | | | | |
| TS02-01 | 200.0 | 200.0 | 1 | 2.00 | 2 |
| TS02-02 | 100.0 | 300.0 | 1 | 4.00 | 4 |
| TS02-03 | 70.0 | 350.0 | 1 | 6.00 | 6 |
| TS02-04 | 25.0 | 175.0 | 1 | 8.00 | 8 |
| TS02-05 | 140.0 | 420.0 | 4 | 4.00 | 16 |
| TS02-06 | 30.0 | 210.0 | 4 | 8.00 | 32 |
| TS02-07 | 75.0 | 150.0 | 16 | 3.00 | 48 |
| TS02-08 | 45.0 | 157.5 | 16 | 4.50 | 72 |
| TS02-09 | 30.0 | 172.5 | 16 | 6.75 | 108 |
| TS02-10 | 40.0 | 162.5 | 32 | 5.06 | 162 |
| **Parent TS: 2163-006, Day 14** | | | | | |
| TS03-01 | 400.0 | 0.0 | 1 | 1.00 | 1 |
| TS03-02 | 100.0 | 100.0 | 1 | 2.00 | 2 |
| TS03-03 | 150.0 | 450.0 | 1 | 4.00 | 4 |
| TS03-04 | 34.0 | 170.0 | 1 | 6.00 | 6 |
| TS03-05 | 50.0 | 350.0 | 1 | 8.00 | 8 |
| TS03-06 | 98.0 | 294.0 | 4 | 4.00 | 16 |
| TS03-07 | 34.0 | 170.0 | 4 | 6.00 | 24 |
| TS03-08 | 90.0 | 112.5 | 16 | 2.25 | 36 |
| TS03-09 | 60.0 | 142.5 | 16 | 3.38 | 54 |
| TS03-10 | 40.0 | 162.5 | 16 | 5.06 | 81 |
| **Parent TS: 2163-010, Day 14** | | | | | |
| TS04-01 | 400.0 | 0.0 | 1 | 1.00 | 1 |
| TS04-02 | 100.0 | 100.0 | 1 | 2.00 | 2 |
| TS04-03 | 150.0 | 450.0 | 1 | 4.00 | 4 |
| TS04-04 | 34.0 | 170.0 | 1 | 6.00 | 6 |
| TS04-05 | 50.0 | 350.0 | 1 | 8.00 | 8 |
| TS04-06 | 98.0 | 294.0 | 4 | 4.00 | 16 |
| TS04-07 | 34.0 | 170.0 | 4 | 6.00 | 24 |
| TS04-08 | 90.0 | 112.5 | 16 | 2.25 | 36 |
| TS04-09 | 60.0 | 142.5 | 16 | 3.38 | 54 |
| TS04-10 | 40.0 | 162.5 | 16 | 5.06 | 81 |
| **Parent TS: 2163-013, Day 180** | | | | | |
| TS05-01 | 400.0 | 0.0 | 1 | 1.00 | 1 |
| TS05-02 | 100.0 | 100.0 | 1 | 2.00 | 2 |
| TS05-03 | 150.0 | 450.0 | 1 | 4.00 | 4 |
| TS05-04 | 34.0 | 170.0 | 1 | 8.00 | 8 |
| TS05-05 | 50.0 | 350.0 | 8 | 2.00 | 16 |
| TS05-06 | 98.0 | 294.0 | 8 | 4.00 | 32 |
| TS05-07 | 34.0 | 170.0 | 8 | 6.00 | 48 |
| TS05-08 | 90.0 | 112.5 | 16 | 4.50 | 72 |
| TS05-09 | 60.0 | 142.5 | 16 | 6.75 | 108 |
| TS05-10 | 40.0 | 162.5 | 32 | 5.06 | 162 |
| **Parent TS: 2163-016, Day 180** | | | | | |
| TS06-01 | 200.0 | 200.0 | 1 | 2.00 | 2 |
| TS06-02 | 50.0 | 150.0 | 1 | 4.00 | 4 |
| TS06-03 | 90.0 | 630.0 | 1 | 8.00 | 8 |
| TS06-04 | 150.0 | 150.0 | 8 | 2.00 | 16 |
| TS06-05 | 100.0 | 300.0 | 8 | 4.00 | 32 |
| TS06-06 | 50.0 | 250.0 | 8 | 6.00 | 48 |
| TS06-07 | 45.0 | 157.5 | 16 | 4.50 | 72 |
| TS06-08 | 30.0 | 172.5 | 16 | 6.75 | 108 |
| TS06-09 | 60.0 | 142.5 | 48 | 3.38 | 162 |
| TS06-10 | 40.0 | 162.5 | 48 | 5.06 | 243 |
| **Parent TS: 2163-023, Day 84** | | | | | |
| TS07-01 | 200.0 | 200.0 | 1 | 2.00 | 4 |
| TS07-02 | 50.0 | 150.0 | 1 | 4.00 | 8 |
| TS07-03 | 90.0 | 630.0 | 1 | 8.00 | 16 |
| TS07-04 | 150.0 | 150.0 | 8 | 2.00 | 32 |
| TS07-05 | 100.0 | 300.0 | 8 | 4.00 | 48 |
| TS07-06 | 50.0 | 250.0 | 8 | 6.00 | 144 |
| TS07-07 | 45.0 | 157.5 | 16 | 4.50 | 216 |
| TS07-08 | 30.0 | 172.5 | 16 | 6.75 | 324 |
| TS07-09 | 60.0 | 142.5 | 48 | 3.38 | 486 |
| TS07-10 | 40.0 | 162.5 | 48 | 5.06 | 729 |
| **Parent TS: 2163-024, Day 84** | | | | | |
| TS08-01 | 100.0 | 300.0 | 1 | 4.00 | 4 |
| TS08-02 | 70.0 | 490.0 | 1 | 8.00 | 8 |
| TS08-03 | 290.0 | 290.0 | 8 | 2.00 | 16 |
| TS08-04 | 65.0 | 195.0 | 8 | 4.00 | 32 |
| TS08-05 | 140.0 | 280.0 | 16 | 3.00 | 48 |
| TS08-06 | 34.0 | 272.0 | 16 | 9.00 | 144 |
| TS08-07 | 30.0 | 172.5 | 32 | 6.75 | 216 |
| TS08-08 | 20.0 | 182.5 | 32 | 10.13 | 324 |
| TS08-09 | 60.0 | 142.5 | 144 | 3.38 | 486 |
| TS08-10 | 40.0 | 162.5 | 144 | 5.06 | 729 |
| **Parent TS: 2163-034, Day 84** | | | | | |
| TS09-01 | 100.0 | 300.0 | 1 | 4.00 | 4 |
| TS09-02 | 75.0 | 525.0 | 1 | 8.00 | 8 |
| TS09-03 | 330.0 | 330.0 | 8 | 2.00 | 16 |
| TS09-04 | 50.0 | 150.0 | 8 | 4.00 | 32 |
| TS09-05 | 185.0 | 370.0 | 16 | 3.00 | 48 |
| TS09-06 | 68.0 | 238.0 | 16 | 4.50 | 72 |
| TS09-07 | 90.0 | 112.5 | 48 | 2.25 | 108 |
| TS09-08 | 60.0 | 142.5 | 48 | 3.38 | 162 |
| TS09-09 | 60.0 | 142.5 | 72 | 3.38 | 243 |
| TS09-10 | 39.6 | 161.15 | 72 | 5.07 | 365 |
| **Parent TS: 2163-071, Day 28** | | | | | |
| TS10-01 | 200.0 | 200.0 | 1 | 2.00 | 2 |
| TS10-02 | 80.0 | 240.0 | 1 | 4.00 | 4 |
| TS10-03 | 60.0 | 420.0 | 1 | 8.00 | 8 |
| TS10-04 | 70.0 | 210.0 | 4 | 4.00 | 16 |
| TS10-05 | 50.0 | 350.0 | 4 | 8.00 | 32 |
| TS10-06 | 50.0 | 250.0 | 8 | 6.00 | 48 |
| TS10-07 | 45.0 | 157.5 | 16 | 4.50 | 72 |
| TS10-08 | 30.0 | 172.5 | 16 | 6.75 | 108 |
| TS10-09 | 60.0 | 142.5 | 48 | 3.38 | 162 |
| TS10-10 | 40.0 | 162.5 | 48 | 5.06 | 243 |
