# Supplementary material for: Development, qualification, and validation of the Filovirus Animal Nonclinical Group anti-Ebola virus glycoprotein immunoglobulin G enzyme-linked immunosorbent assay for human serum samples
Source: PLoS One. 2019 Apr 18;14(4):e0215457. doi: 10.1371/journal.pone.0215457 (PMC6472792; doi:10.1371/journal.pone.0215457)
Supplement: S12 Table — (DOCX) [file pone.0215457.s022.docx]

Table S12. Parent test samples, dilution factors, and starting dilutions for validation test samples.

| **VTS #** | **Immune Specimen** | **Naïve Specimen** | **Final Dilution Factor (Spike)** | **VTS Starting Dilution** |
| --- | --- | --- | --- | --- |
| 1 | W092115060057-A | NA | 1:1 | 1:200 |
| 2 |  | NA | - | 1:400 |
| 3 |  | NA | - | 1:100 |
| 4 |  | BMI530 | 1:2 | 1:100 |
| 5 |  |  | 1:4 | 1:50 |
| 6 |  |  | 1:8 | 1:50 |
| 7 |  |  | 1:16 | 1:50 |
| 8 |  |  | 1:48 | 1:50 |
| 9 |  |  | 1:96 | 1:50 |
| 10 |  |  | 1:192 | 1:50 |
| 11 | W092115060064-A | NA | 1:1 | 1:100 |
| 12 |  | BMI530 | 1:2 | 1:100 |
| 13 |  | - | - | 1:200 |
| 14 |  | - | - | 1:50 |
| 15 |  | BMI530 | 1:4 | 1:50 |
| 16 |  |  | 1:8 | 1:50 |
| 17 |  |  | 1:16 | 1:50 |
| 18 |  |  | 1:48 | 1:50 |
| 19 |  |  | 1:96 | 1:50 |
| 20 |  |  | 1:192 | 1:50 |
| 21 | 650062386624 | NA | 1:1 | 1:200 |
| 22 |  | - | - | 1:400 |
| 23 |  | - | - | 1:100 |
| 24 |  | BMI530 | 1:3 | 1:100 |
| 25 |  |  | 1:9 | 1:50 |
| 26 |  |  | 1:27 | 1:50 |
| 27 |  |  | 1:54 | 1:50 |
| 28 |  |  | 1:108 | 1:50 |
| 29 |  |  | 1:216 | 1:50 |
| 30 |  |  | 1:432 | 1:50 |
| 31 |  | NA | 1:1 | 1:600 |
| 32 |  | - | - | 1:1200 |
| 33 |  | - | - | 1:300 |
| 34 |  | BMI530 | 1:3 | 1:200 |
| 35 |  |  | 1:9 | 1:100 |
| 36 |  |  | 1:27 | 1:50 |
| 37 |  |  | 1:54 | 1:50 |
| 38 |  |  | 1:324 | 1:50 |
| 39 |  |  | 1:432 | 1:50 |
| 40 |  |  | 1:864 | 1:50 |

| **VTS #** | **Immune Specimen** | **Naïve Specimen** | **Final Dilution Factor (Spike)** | **VTS Starting Dilution** |
| --- | --- | --- | --- | --- |
| 41 | 10113200-03 | NA | 1:1 | 1:200 |
| 42 |  | BMI530 | 1:2 | 1:100 |
| 43 |  | - | - | 1:200 |
| 44 |  | - | - | 1:50 |
| 45 |  | BMI530 | 1:4 | 1:50 |
| 46 |  |  | 1:8 | 1:50 |
| 47 |  |  | 1:16 | 1:50 |
| 48 |  |  | 1:48 | 1:50 |
| 49 |  |  | 1:96 | 1:50 |
| 50 |  |  | 1:192 | 1:50 |
| 51 |  | NA | 1:1 | 1:400 |
| 52 |  | BMI530 | 1:2 | 1:200 |
| 53 |  | - | - | 1:400 |
| 54 |  | - | - | 1:100 |
| 55 |  | BMI530 | 1:4 | 1:50 |
| 56 |  |  | 1:8 | 1:50 |
| 57 |  |  | 1:16 | 1:50 |
| 58 |  |  | 1:48 | 1:50 |
| 59 |  |  | 1:96 | 1:50 |
| 60 |  |  | 1:192 | 1:50 |
| 61 | 3344.013.D180 | NA | NA | 1:400 |
| 62 |  | - | - | 1:800 |
| 63 |  | - | - | 1:200 |
| 64 |  | BMI530 | 1:2 | 1:100 |
| 65 |  |  | 1:4 | 1:50 |
| 66 |  |  | 1:8 | 1:50 |
| 67 |  |  | 1:16 | 1:50 |
| 68 |  |  | 1:48 | 1:50 |
| 69 |  |  | 1:96 | 1:50 |
| 70 |  |  | 1:192 | 1:50 |
| 71 | 3344.043.D84 | NA | 1:1 | 1:500 |
| 72 |  | BMI530 | 1:3 | 1:100 |
| 73 |  | - | - | 1:200 |
| 74 |  | - | - | 1:50 |
| 75 |  | BMI530 | 1:9 | 1:50 |
| 76 |  |  | 1:27 | 1:50 |
| 77 |  |  | 1:54 | 1:50 |
| 78 |  |  | 1:108 | 1:50 |
| 79 |  |  | 1:216 | 1:50 |
| 80 |  |  | 1:432 | 1:50 |

| **VTS #** | **Immune Specimen** | **Naïve Specimen** | **Final Dilution Factor (Spike)** | **VTS Starting Dilution** |
| --- | --- | --- | --- | --- |
| 81 | BMIZAIRE112 | NA | 1:1 | 1:500 |
| 82 |  | - | - | 1:1000 |
| 83 |  | - | - | 1:250 |
| 84 |  | BMI530 | 1:3 | 1:200 |
| 85 |  |  | 1:9 | 1:100 |
| 86 |  |  | 1:27 | 1:50 |
| 87 |  |  | 1:54 | 1:50 |
| 88 |  |  | 1:324 | 1:50 |
| 89 |  |  | 1:432 | 1:50 |
| 90 |  |  | 1:864 | 1:50 |
| 91 | BMIZAIRE113 | NA | 1:1 | 1:300 |
| 92 |  | BMI530 | 1:3 | 1:100 |
| 93 |  | - | - | 1:200 |
| 94 |  | - | - | 1:50 |
| 95 |  | BMI530 | 1:9 | 1:50 |
| 96 |  |  | 1:27 | 1:50 |
| 97 |  |  | 1:54 | 1:50 |
| 98 |  |  | 1:108 | 1:50 |
| 99 |  |  | 1:216 | 1:50 |
| 100 |  |  | 1:432 | 1:50 |
| 101 | W092115060071-A | BMI530 | 1:5 | 1:50 |
| 102 |  | 23 79329 (Male) | 1:5 | 1:50 |
| 103 |  | 23 79327 (Male) | 1:5 | 1:50 |
| 104 |  | 88 21595 (Female) | 1:5 | 1:50 |
| 105 |  | 88 22472 (Female) | 1:5 | 1:50 |
| 106 |  | 88 22458 (Female) | 1:5 | 1:50 |
| 107 |  | BMI530 | 1:50 | 1:50 |
| 108 |  | 23 79329 (Male) | 1:50 | 1:50 |
| 109 |  | 23 79327 (Male) | 1:50 | 1:50 |
| 110 |  | 88 21595 (Female) | 1:50 | 1:50 |
| 111 |  | 88 22472 (Female) | 1:50 | 1:50 |
| 112 |  | 88 22458 (Female) | 1:50 | 1:50 |

| **VTS #** | **Immune Specimen** | **Naïve Specimen** | **Final Dilution Factor (Spike)** | **VTS Starting Dilution** |
| --- | --- | --- | --- | --- |
| 113 | 650062384124 | BMI530 | 1:5 | 1:100 |
| 114 |  | 23 79329 (Male) | 1:5 | 1:100 |
| 115 |  | 23 79327 (Male) | 1:5 | 1:100 |
| 116 |  | 88 21595 (Female) | 1:5 | 1:100 |
| 117 |  | 88 22472 (Female) | 1:5 | 1:100 |
| 118 |  | 88 22458 (Female) | 1:5 | 1:100 |
| 119 |  | BMI530 | 1:50 | 1:50 |
| 120 |  | 23 79329 (Male) | 1:50 | 1:50 |
| 121 |  | 23 79327 (Male) | 1:50 | 1:50 |
| 122 |  | 88 21595 (Female) | 1:50 | 1:50 |
| 123 |  | 88 22472 (Female) | 1:50 | 1:50 |
| 124 |  | 88 22458 (Female) | 1:50 | 1:50 |
| 125 | 10131804-03 | BMI530 | 1:5 | 1:200 |
| 126 |  | 23 79329 (Male) | 1:5 | 1:200 |
| 127 |  | 23 79327 (Male) | 1:5 | 1:200 |
| 128 |  | 88 21595 (Female) | 1:5 | 1:200 |
| 129 |  | 88 22472 (Female) | 1:5 | 1:200 |
| 130 |  | 88 22458 (Female) | 1:5 | 1:200 |
| 131 |  | BMI530 | 1:50 | 1:50 |
| 132 |  | 23 79329 (Male) | 1:50 | 1:50 |
| 133 |  | 23 79327 (Male) | 1:50 | 1:50 |
| 134 |  | 88 21595 (Female) | 1:50 | 1:50 |
| 135 |  | 88 22472 (Female) | 1:50 | 1:50 |
| 136 |  | 88 22458 (Female) | 1:50 | 1:50 |
| 137 | W092115060101-C | Hemoglobin (High) | 1:20 | 1:50 |
| 138 |  | Hem (Low) + BMI530 | 1:20 | 1:50 |
| 139 |  | Hem Mock (BMI530) | 1:20 | 1:50 |
| 140 |  | Albumin (50 mg/mL) | 1:1 | 1:100 |
| 141 |  | Albumin Mock (BMI530) | 1:1 | 1:100 |
| 142 |  | Triglycerides (5 mg/mL) | 1:1.05 | 1:100 |
| 143 |  | Triglycerides Mock (BMI530) | 1:1.05 | 1:100 |
| 144 |  | Bilirubin (0.15 mg/mL) | 1:1.05 | 1:100 |
| 145 |  | Bilirubin Mock (1:20 DMSO) | 1:1.05 | 1:100 |

| **VTS #** | **Immune Specimen** | **Naïve Specimen** | **Final Dilution Factor (Spike)** | **VTS Starting Dilution** |
| --- | --- | --- | --- | --- |
| 146 | 650041667124 | Hemoglobin (High) | 1:20 | 1:50 |
| 147 |  | Hem (Low) + BMI530 | 1:20 | 1:50 |
| 148 |  | Hem Mock (BMI530) | 1:20 | 1:50 |
| 149 |  | Albumin (50 mg/mL) | 1:1 | 1:100 |
| 150 |  | Albumin Mock (BMI530) | 1:1 | 1:100 |
| 151 |  | Triglycerides (5 mg/mL) | 1:1.05 | 1:100 |
| 152 |  | Triglycerides Mock (BMI530) | 1:1.05 | 1:100 |
| 153 |  | Bilirubin (0.15 mg/mL) | 1:1.05 | 1:100 |
| 154 |  | Bilirubin Mock (1:20 DMSO) | 1:1.05 | 1:100 |
| 155 | BMIZAIRE114 | Hemoglobin (High) | 1:20 | 1:50 |
| 156 |  | Hem (Low) + BMI530 | 1:20 | 1:50 |
| 157 |  | Hem Mock (BMI530) | 1:20 | 1:50 |
| 158 |  | Albumin (50 mg/mL) | 1:1 | 1:100 |
| 159 |  | Albumin Mock (BMI530) | 1:1 | 1:100 |
| 160 |  | Triglycerides (5 mg/mL) | 1:1.05 | 1:100 |
| 161 |  | Triglycerides Mock (BMI530) | 1:1.05 | 1:100 |
| 162 |  | Bilirubin (0.15 mg/mL) | 1:1.05 | 1:100 |
| 163 |  | Bilirubin Mock (1:20 DMSO) | 1:1.05 | 1:100 |
| 164 | BMI530 | Hemoglobin (High) | 1:20 | 1:50 |
| 165 |  | Hem (Low) + BMI530 | 1:20 | 1:50 |
| 166 |  | Hem Mock (BMI530) | 1:20 | 1:50 |
| 167 |  | Albumin (50 mg/mL) | 1:1 | 1:50 |
| 168 |  | Albumin Mock (BMI530) | 1:1 | 1:50 |
| 169 |  | Triglycerides (5 mg/mL) | 1:1.05 | 1:50 |
| 170 |  | Triglycerides Mock (BMI530) | 1:1.05 | 1:50 |
| 171 |  | Bilirubin (0.15 mg/mL) | 1:1.05 | 1:50 |
| 172 |  | Bilirubin Mock (1:20 DMSO) | 1:1.05 | 1:50 |
| 173 | W092115060052-A | Mock | 1:1 | 1:200 |
| 174 |  | rGP - 25 µg/mL | 1:1 | 1:200 |
| 175 |  | CMV - 25 µg/mL | 1:1 | 1:200 |
| 176 | W092115060057-B | Mock | 1:1 | 1:200 |
| 177 |  | rGP - 25 µg/mL | 1:1 | 1:200 |
| 178 |  | CMV - 25 µg/mL | 1:1 | 1:200 |
| 179 | 650041667024 | Mock | 1:1 | 1:100 |
| 180 |  | rGP - 25 µg/mL | 1:1 | 1:100 |
| 181 |  | CMV - 25 µg/mL | 1:1 | 1:100 |
| 182 | 650062385224 | Mock | 1:1 | 1:400 |
| 183 |  | rGP - 25 µg/mL | 1:1 | 1:400 |
| 184 |  | CMV - 25 µg/mL | 1:1 | 1:400 |

| **VTS #** | **Immune Specimen** | **Naïve Specimen** | **Final Dilution Factor (Spike)** | **VTS Starting Dilution** |
| --- | --- | --- | --- | --- |
| 185 | 10113735-03 | Mock | 1:1 | 1:100 |
| 186 |  | rGP - 25 µg/mL | 1:1 | 1:100 |
| 187 |  | CMV - 25 µg/mL | 1:1 | 1:100 |
| 188 | 10114436-03 | Mock | 1:1 | 1:100 |
| 189 |  | rGP - 25 µg/mL | 1:1 | 1:100 |
| 190 |  | CMV - 25 µg/mL | 1:1 | 1:100 |
| 191 | 3344.042.D56 | Mock | 1:1 | 1:100 |
| 192 |  | rGP - 25 µg/mL | 1:1 | 1:100 |
| 193 |  | CMV - 25 µg/mL | 1:1 | 1:100 |
| 194 | 3344.044.D84 | Mock | 1:1 | 1:100 |
| 195 |  | rGP - 25 µg/mL | 1:1 | 1:100 |
| 196 |  | CMV - 25 µg/mL | 1:1 | 1:100 |
| 197 | C1703090460 | Mock | 1:1 | 1:50 |
| 198 |  | rGP - 25 µg/mL | 1:1 | 1:50 |
| 199 |  | CMV - 25 µg/mL | 1:1 | 1:50 |
| 200 | C1703087970 | Mock | 1:1 | 1:50 |
| 201 |  | rGP - 25 µg/mL | 1:1 | 1:50 |
| 202 |  | CMV - 25 µg/mL | 1:1 | 1:50 |
| 203 | W092115060052-B | NA | 1:1 | 1:200 |
| 204 |  | NA | 1:1 | 1:100 |
| 205 |  | NA | 1:1 | 1:100 |
| 206 | W092115060138-A | NA | 1:1 | 1:100 |
| 207 | 650050315424 | NA | 1:1 | 1:200 |
| 208 | 650062384524 | NA | 1:1 | 1:200 |
| 209 |  | NA | 1:1 | 1:300 |
| 210 |  | NA | 1:1 | 1:300 |
| 211 | 10113168-03 | NA | 1:1 | 1:50 |
| 212 | 10113528-03 | NA | 1:1 | 1:50 |
| 213 | 10113989-03 | NA | 1:1 | 1:50 |
| 214 | 10130517-03 | NA | 1:1 | 1:50 |
| 215 | 3344.008.D14 | NA | 1:1 | 1:50 |
| 216 | 3344.011.D180 | NA | 1:1 | 1:50 |
| 217 | 3344.014.D14 | NA | 1:1 | 1:50 |
| 218 | 3344.020.D56 | NA | 1:1 | 1:50 |
| 219 | 3344.025.D14 | NA | 1:1 | 1:50 |
| 220 | 3344.037.D14 | NA | 1:1 | 1:50 |
| 221 | C1703024660 | NA | 1:1 | 1:50 |
| 222 | C1703089620 | NA | 1:1 | 1:50 |
| 223 | C1703186220 | NA | 1:1 | 1:50 |
| 224 | C1703042080 | NA | 1:1 | 1:50 |
| 225 | C1703060550 | NA | 1:1 | 1:50 |
| **VTS #** | **Immune Specimen** | **Naïve Specimen** | **Final Dilution Factor (Spike)** | **VTS Starting Dilution** |
| 226 | C1703068810 | NA | 1:1 | 1:50 |
| 227 | C1703187960 | NA | 1:1 | 1:50 |
| 228 | BMIZAIRE117 | NA | 1:1 | 1:2300 |
| 229 | BMIZAIRE117 | BMI530 | 1:1.25 | 1:180 |
| 230 | BMIZAIRE117 | BMI530 | 1:1.56 | 1:1500 |
| 231 | BMIZAIRE117 | BMI530 | 1:1.95 | 1:1200 |
| 232 | BMIZAIRE117 | BMI530 | 1:2.44 | 1:900 |
| 233 | BMIZAIRE117 | BMI530 | 1:3.05 | 1:700 |
| 234 | BMIZAIRE117 | BMI530 | 1:3.81 | 1:600 |
| 235 | BMIZAIRE117 | BMI530 | 1:7.63 | 1:300 |
| 236 | BMIZAIRE117 | BMI530 | 1:15.26 | 1:100 |
| 237 | BMIZAIRE117 | BMI530 | 1:30.52 | 1:100 |
| 238 | BMIZAIRE118 | NA | 1:1 | 1:2000 |
| 239 | BMIZAIRE118 | BMI530 | 1:1.25 | 1:1600 |
| 240 | BMIZAIRE118 | BMI530 | 1:1.56 | 1:1300 |
| 241 | BMIZAIRE118 | BMI530 | 1:1.95 | 1:1000 |
| 242 | BMIZAIRE118 | BMI530 | 1:2.44 | 1:800 |
| 243 | BMIZAIRE118 | BMI530 | 1:3.05 | 1:700 |
| 244 | BMIZAIRE118 | BMI530 | 1:3.81 | 1:500 |
| 245 | BMIZAIRE118 | BMI530 | 1:7.63 | 1:300 |
| 246 | BMIZAIRE118 | BMI530 | 1:15.26 | 1:100 |
| 247 | BMIZAIRE118 | BMI530 | 1:30.52 | 1:100 |
| 248 | BMIZAIRE119 | NA | 1:1 | 1:2000 |
| 249 | BMIZAIRE119 | BMI530 | 1:1.25 | 1:1600 |
| 250 | BMIZAIRE119 | BMI530 | 1:1.56 | 1:1300 |
| 251 | BMIZAIRE119 | BMI530 | 1:1.95 | 1:1000 |
| 252 | BMIZAIRE119 | BMI530 | 1:2.44 | 1:800 |
| 253 | BMIZAIRE119 | BMI530 | 1:3.05 | 1:700 |
| 254 | BMIZAIRE119 | BMI530 | 1:3.81 | 1:500 |
| 255 | BMIZAIRE119 | BMI530 | 1:7.63 | 1:300 |
| 256 | BMIZAIRE119 | BMI530 | 1:15.26 | 1:100 |
| 257 | BMIZAIRE119 | BMI530 | 1:30.52 | 1:100 |
